# Crosstalk Between Culturomics and Microbial Profiling of Egyptian Mongoose (*Herpestes ichneumon*) Gut Microbiome

**DOI:** 10.3390/microorganisms8060808

**Published:** 2020-05-27

**Authors:** André C. Pereira, Victor Bandeira, Carlos Fonseca, Mónica V. Cunha

**Affiliations:** 1 National Institute for Agrarian and Veterinary Research (INIAV, IP), Wildlife, Hunting and Biodiversity R&D Unit, 2780-157 Oeiras, Portugal; andre.c.pereira94@gmail.com; 2 Centre for Ecology, Evolution and Environmental Changes (cE3c), Faculdade de Ciências da Universidade de Lisboa, 1749-016 Lisboa, Portugal; 3 Biosystems & Integrative Sciences Institute (BioISI), Faculdade de Ciências da Universidade de Lisboa, 1749-016 Lisboa, Portugal; 4 Departamento de Biologia & CESAM, Universidade de Aveiro, 3810-193 Aveiro, Portugal; victor.bandeira@ua.pt (V.B.); cfonseca@ua.pt (C.F.)

**Keywords:** Egyptian mongoose, gut microbiota, culturomics, microbial profiling

## Abstract

Recently, we unveiled taxonomical and functional differences in Egyptian mongoose (*Herpestes ichneumon*) gut microbiota across sex and age classes by microbial profiling. In this study, we generate, through culturomics, extended baseline information on the culturable bacterial and fungal microbiome of the species using the same specimens as models. Firstly, this strategy enabled us to explore cultivable microbial community differences across sexes and to ascertain the influence exerted by biological and environmental contexts of each host in its microbiota signature. Secondly, it permitted us to compare the culturomics and microbial profiling approaches and their ability to provide information on mongoose gut microbiota. In agreement with microbial profiling, culturomics showed that the core gut cultivable microbiota of the mongoose is dominated by *Firmicutes* and, as previously found, is able to distinguish sex- and age class-specific genera. Additional information could be obtained by culturomics, with six new genera unveiled. Richness indices and the Shannon index were concordant between culture-dependent and culture-independent approaches, highlighting significantly higher values when using microbial profiling. However, the Simpson index underlined higher values for the culturomics-generated data. These contrasting results were due to a differential influence of dominant and rare taxa on those indices. Beta diversity analyses of culturable microbiota showed similarities between adults and juveniles, but not in the data series originated from microbial profiling. Additionally, whereas the microbial profiling indicated that there were several bioenvironmental features related to the bacterial gut microbiota of the Egyptian mongoose, a clear association between microbiota and bioenvironmental features could not be established through culturomics. The discrepancies found between the data generated by the two methodologies and the underlying inferences, both in terms of β-diversity and role of bioenvironmental features, confirm that culture-independent, sequence-based methods have a higher ability to assess, at a fine scale, the influence of abiotic and biotic factors on the microbial community composition of mongoose’ gut. However, when used in a complementary perspective, this knowledge can be expanded by culturomics.

## 1. Introduction

The vertebrate gastrointestinal (GI) tract is a complex ecosystem that is the habitat of an enormous density and diversity of microorganisms, containing mostly bacteria, but also archaea, fungi, protozoa, and viruses [1,2]. In carnivores, microbial density and diversity differ within gut sections, with the main concentration of bacteria found in the large intestine, dominated by strict anaerobes [3]. A mammal’s GI tract is usually dominated by members of *Firmicutes*, *Bacteroidetes*, and *Proteobacteria* phyla [4], however, there are a variety of effects that can alter this equilibrium [5]. Accumulated evidence indicates that the host diet has such a deep effect on the gut microbiota that it has resulted in an evolutionary divergence between carnivores and herbivores, leading to two distinct gut types, with an increase in microbiota diversity from carnivores to herbivores, and the dichotomy between foregut fermenters vs. hindgut fermenters herbivores [4,6]. Additionally, gut microbiota plays a role in host energy uptake [7], nutrient absorption, elimination of toxic metabolic by-products, production of vitamins, and regulation of the xenobiotic metabolism [5]. The gut microbiota has been shown to modulate the host immune system, by attenuating inflammatory responses and increasing resistance to pathogenic bacteria; by assisting in the development and maturation of the host intestinal mucosal and systemic immune systems and by aiding in the development and function of the brain and modulation of behavior [5]. Microbiota attained primarily in life is inherited from the mother or social contacts. Hosts within more interconnected social groups show higher frequencies of social contact and less inter-individual variation in gut microbial community structure [8]. More recently, host genetics has been associated with the taxonomic structure of the gut microbiota, since intraspecific differences have been observed [9].

In previous work, we investigated the gut microbiota of the Egyptian mongoose (*Herpestes ichneumon*, Linnaeus, 1758), a medium-sized mammalian carnivore from the *Herpestidae* family, by applying microbial profiling to the 16S rRNA gene sequences of twenty paired male (n = 10) and female (n = 10) intestinal samples [10]. The core gastrointestinal microbiota of this population was dominated by *Firmicutes* (86%), *Actinobacteria* (6%), *Fusobacteria* (3%), *Proteobacteria* (3%), and *Bacteroidetes* (1%), with *Clostridioides* (25%) and *Clostridium* (19%) being the most abundant genera. *Bacteroides*, *Carnobacterium*, *Cetobacterium*, *Enterococcus*, *Fusobacterium*, *Lactobacillus*, *Romboutsia*, and *Sporosarcina* genera were uniquely found in adults and *Coprococcus*, *Coriobacteriaceae*_uc, *Eubacterium*_uc, *Hathewaya*, and *Slackia* genera were uniquely found in nonadults. When comparing gut bacterial communities across sexes, four genera were exclusive in females (*Bacteroides*, *Carnobacterium*, *Cetobacterium*, and *Enterococcus*) and six were found uniquely in males (*Coprococcus*, *Faecalimonas*, *Romboutsia*, *Slackia*, *Sporosarcina*, and *Staphylococcus*). However, no statistically significant differences were found in α- and β-diversity analyses across sex or age classes. Additionally, the influence of several biotic and abiotic factors on gut microbiota was tested, with reptile and mammal stomach contents and all biometric factors evaluated as influential, together with land-use, temperature, rainfall, river net, and road net.

Although culture-independent methods have become central for microbiota studies, several considerable limitations have been reported related to bias across studies, for example, different DNA extraction methods, target regions, and lack of sensitivity to distinguish some bacterial genera [11]. However, in the last decade, a new high-throughput methodology has been developed and has been optimized to isolate and identify the culturable microbiota culturomics [12]. This approach used several enrichment and inoculation media, together with several incubation conditions, to culture a wide range of microorganisms present in the sample. The isolation was coupled with taxonomical identification by 16S rRNA gene sequencing of the entire V1–V9 hypervariable regions or by matrix-assisted laser desorption ionization time-of-flight mass spectrometry (MALDI-TOF MS) [13]. There are several major advantages in this method which include: (1) the ability to mimic different natural conditions present in the gastrointestinal tract, (2) the detection of taxa present in low abundance, (3) the evaluation of cell viability, and (4) the possibility to use the obtained isolates in downstream studies [14]. Moreover, the complementarity between these two approaches was demonstrated, since only 15% of bacterial species are concomitantly detected by both approaches when used in parallel [12].

Taking the culturomics’ advantages into consideration, in this work, we aimed to compare both techniques in their ability to obtain taxonomical information on the gut microbiota of the Egyptian mongoose, a species with opportunistic feeding behavior, consuming mostly rabbits, but also reptiles, other small mammals, amphibians, birds, crayfish, eggs, or carrion [15]. Although this species originated on the African continent, it has expanded into the Mediterranean Middle East and southern Turkey [16]. In the Iberian Peninsula, the Egyptian mongoose was conventionally considered as an introduced species during the Muslim invasions [17,18]. However, the historical process that led to the Egyptian mongoose colonization of Iberia is still under discussion. Gaubert et al. (2011) proposed that mongooses reached Iberia during the Middle to Late Pleistocene [19]. More recently, Detry et al. (2018) suggested that this herpestid could have been introduced by the Romans, during their presence in Hispania [20]. 

In the recent past, the Egyptian mongoose was restricted in Portugal to the south of the Tagus River [21], nonetheless, in the last three decades, the species gradually expanded into central and northeastern regions [22]. This expansion was mostly driven by land-use changes in shrub-dominated ecosystems, forest clearing, agricultural practices, and climate change [23]. Evidence of both sexual and regional dimorphism in the body size of Egyptian mongoose adults in Portugal has been found. This dimorphism probably results from differences in feeding habits, leading to southern male adults that are larger and heavier [24], and possibly leading to functional differences. 

For the purpose of this study, we thoroughly investigated the gut microbiota of the same previously studied twenty Egyptian mongoose specimens sampled in South Portugal, for whom bioecological data was available. The aims were as follows: (1) to compare culturomics and microbial profiling in their ability to assess taxonomical information and identify sex- and age class-related differences, as well as the relationship between bioenvironmental features and individual gut microbiota of these specimens; (2) to highlight the main differences in results obtained from the two approaches; (3) to ascertain the information that can be added by culturomics; and (4) to expose the limits of culturomics.

## 2. Materials and Methods 

### 2.1. Egyptian Mongoose Specimens

Egyptian mongoose carcasses (ten male and ten female) obtained from legal predator density control actions were opportunistically used for this work. They were harvested from the Baixo Alentejo region, south of the Tagus River, from a landscape predominated by agroforestry and agriculture. These carcasses were donated by hunters for scientific purposes and no animals were sacrificed for this study. After death, animal carcasses were frozen at −20 °C until necropsy. Information regarding sex, age class, geographic location, land-use, and stomach content at the time of death was registered. Age class distribution was 16 adults and four nonadults (two subadults and two juveniles). The selected animals had the same stomach content at death, mostly composed of mammal and egg items [15].

Mongooses were subjected to necropsy (within less than 3 days after death) and specimen collection by pathologists at the necropsy facilities of the National Institute for Agrarian and Veterinary Research (INIAV, IP), the National Reference Laboratory for Animal Health. There were no signs of putrefaction or disease detected. The abdominal cavity of each specimen was opened and the intestines isolated. Solid intestinal content (colon) was collected from each animal using a sterile feces collection tube and immediately processed for further analyses. 

### 2.2. Bacteriological Culture

Sample storage, enrichment, pasteurization, and inoculation were performed as described elsewhere [25]. The fecal samples (2.5 g) were homogenized and divided into two parts, 0.5 g were used in the previous microbial profiling work [10] and 2 g were used in this study. The samples (2 g) were divided into two equal parts, homogenized in buffered peptone water (10 mL), and incubated at 37 °C for 24 h under aerobic (with O2) and anaerobic (without O2) conditions. Following, 1 mL of each enriched sample was pasteurized at 80 °C for 15 min and plated onto Yeast extract, casitone, and fatty acid (YCFA) agar supplemented with sodium taurocholate (YCFA P) [26] and incubated in the same oxygenic condition. The pasteurization followed by growth on sodium taurocholate enriched media enables the killing of vegetative cells and the germination of bacterial spores, respectively. The rest of each enriched sample incubated under anaerobic conditions was plated onto YCFA agar (YCFA) [26] and incubated in anaerobic conditions. The use of this medium under anaerobiosis allows the detection of vegetative anaerobic bacteria. Additionally, the rest of each enriched sample incubated under aerobic conditions was plated onto YCFA (detection of vegetative aerobic bacteria), MacConkey medium (detection of coliforms), potato dextrose agar supplemented with chloramphenicol (PDA + CHLO) (detection of fungi), extended-spectrum beta-lactamase (ESBL) chromogenic medium, with (ESBL with AS) and without (ESBL without AS) ESBL supplement of antibiotics, and Brilliance ESBL medium (Brilliance), following incubation. ESBL w/AS and Brilliance allow the detection of ESBL-producing bacteria. All media were incubated at 37 °C for 24 h, except YCFA P, YCFA, and PDA + CHLO media that were incubated for 72 h. Each medium was inoculated with three different enrichment dilutions. Colony-forming units per gram of wet fecal weight (CFU/g) were determined. 

### 2.3. Purification and Presumptive Identification of Isolates

For purification of isolates, five isolated colonies from each different morphology were picked from different dilutions of all media. The 1500 purified bacterial isolates were characterized in terms of gram character, endospore formation, and the presence of catalase and cytochrome c oxidase enzymes. In ESBL chromogenic medium (Condalab, Madrid, Spain) and Brilliance ESBL medium (Oxoid, Lenexa, KS, USA), colony color enabled the differentiation of bacterial isolates, according to specific information provided by the manufacturers. The 30 isolated fungi were all morphologically characterized both at the macroscopic and microscopic levels.

Following the previous characterizations, all isolates were grouped into morpho-physiological types (MT) described elsewhere [25] and as follows: MT-I to MT-XI for bacterial isolates (Supplementary Figure S1), MT-XII for yeasts (non-filamentous fungi), and MT-XIII for filamentous fungi. Additionally, MT-Vibrium and MT-Others were created, the first to place a vibrium-like isolate and the second to place atypical, unidentifiable isolates.

### 2.4. Molecular Identification and Molecular Fingerprinting of Bacterial Isolates

To analyze the intraspecific polymorphism present in the overall bacterial populations which would provide a framework for selecting the isolates subjected to molecular identification, 500 bacterial isolates were selected for molecular fingerprinting (random amplified polymorphic DNA (RAPDs)) based on MT, solid media, and host criteria. Total DNA extraction was performed by direct boiling of 2 to 3 colonies suspended in TE buffer, at 95 °C for 15 min, centrifuging, and collection of the supernatant into a clear microtube, and stored at −20 °C.

Two primers were used to perform molecular fingerprinting of the bacterial isolates, M13 (5’-GAG GGT GGC GGT TCT-3’) [27] and PH (5’-AAG GAG GTG ATC CAG CCG CA-3’) [28]. The preparation of PCR reaction mixes, cycling conditions, and product resolution was performed as described elsewhere [25]. Briefly, 7.5 μL of NZYTaq II 2× Green Master Mix (NZYTech, Lisbon, Portugal), 0.2 mM of each primer (Invitrogen, Waltham, MA, USA), and 5 μL of DNA were used in a final volume of 15 μL. The PCR cycling conditions for M13 consisted of 94 °C for 5 min, 40 cycles of 60 s at 94 °C, 3 min at 40 °C, 120 s at 72 °C, and a final step of 7 min at 72 °C. The PCR cycling conditions for PH consisted of 95 °C for 3 min, 35 cycles of 30 s at 94 °C, 30 s at 35 °C, 3 min at 72 °C, plus an additional step at 72 °C for 5 min. The PCR products were resolved by 1.5% (w/v) agarose gel, 90 V, 3 h.

A total of 18 randomly selected duplicates for M13 typing and 22 for PH typing were used as a measure of reproducibility. The similarity between each pair of duplicates was analyzed by hierarchical numerical methods using Pearson correlation similarity and unweighted pair group method with arithmetic average (UPGMA) clustering (BioNumerics version 4.0, Applied Maths, Sint-Martens-Latem, Belgium). The reproducibility value was determined as the average value for all pairs of duplicates. On the basis of the fingerprints, the strain relationship was obtained on the dendrograms computed with the Pearson correlation coefficient and the UPGMA as the agglomerative clustering algorithm, using 70% similarity as the cutoff value for cluster formation.

A panel of 139 isolates (one random isolate from each cluster) was selected for 16S rRNA gene sequence analyses to accomplish molecular identification. A PCR was performed using primers, reaction mixes, and cycling conditions as reported [25]. Briefly, PCR was performed using 63f (5′-CAG GCC TAA CAC ATG CAA GTC-3′) and 1387r (5′-GGG CGG WGT GTA CAA GGC-3′) primers, in a final volume of 25 μL with 0.2 mM from each primer (Invitrogen, Waltham, MA, USA), 12.5 μL NZYTaq II 2× Green Master Mix, and 5 μL of DNA. The thermal profile of the PCR consisted of 5 min at 95 °C, 30 cycles of 45 s at 95 °C, 45 s at 55 °C, 120 s at 72 °C, and a final step of 7 min at 72 °C. The selected pair of primers led to the amplification of a fragment encompassing all hypervariable regions (V1–V9). The PCR products with the expected size (approximately 1500 bp) were quantified using a Qubit fluorometer (Invitrogen, Waltham, MA, USA), following the manufacturer’s instructions. Samples were commercially sequenced by the Sanger sequencing technique using 63f primer (GATC Biotech AG, Konstanz, Germany).

### 2.5. Molecular Identification of Fungi

Six morphologically different fungi isolates were selected for genomic identification through sequencing. Yeast DNA was extracted using the direct boiling method previously described, and filamentous fungi DNA were extracted using an NZY Plant/Fungi gDNA Isolation kit (NZYTech, Lisbon, Portugal), following the manufacturer’s instructions.

Amplification of the D1/D2 domain region of the 26S rRNA gene in yeast [29] and the internal transcribed spacer (ITS) region in filamentous fungi were performed [30], using primers, reaction mixes, and cycling conditions as indicated elsewhere [25]. Briefly, the D1/D2 domain region was amplified using NL-1 (5’-GCA TAT CAA TAA GCG GAG GAA AAG-3’) and NL-4 (5’-GGT CCG TGT TTC AAG ACG G-3’) primers and the ITS region was amplified using ITS5 (5’-GGA AGT AAA AGT CGT AAC AAG G-3’) and ITS4 (5’-TCC TCC GCT TAT TGA TAT GC-3’) primers. A final volume of 25 μL was used containing 0.2 mM of each pair of primers (Invitrogen, Waltham, MA, USA), 12.5 μL NZYTaq II 2× Green Master Mix, and 5 μL of DNA. The PCR amplification consisted of 3 min at 95 °C, 35 cycles of 30 s at 94 °C, 30 s at 55 °C, 30 s at 72 °C, and a final step of 10 min at 72 °C.

The PCR products with the expected size (approximately 650 bp and between 600 and 800 bp, respectively) were extracted using QIAquick Gel Extraction Kit (QIAGEN, Venlo, The Netherlands), according to the manufacturer’s instructions. DNA was quantified using a Qubit fluorometer, following the manufacturer’s instructions. Samples were commercially sequenced by the Sanger sequencing technique (GATC Biotech AG, Konstanz, Germany).

### 2.6. Homology Searches for Gene-Based Identification of Isolates

Electropherograms were manually analyzed and corrected when necessary and undetermined nucleotides were designated as N. All 16S rRNA gene partial sequences generated were located in the early region of the gene (V1–V3), which is informative for the identification of most genera since it is a highly polymorphic moiety [31]. The obtained sequences were compared with those available in the GenBank databases using the BLASTN program through the National Center for Biotechnology Information (NCBI) server. Comparisons were performed using the default parameters. The top three best matches were annotated with taxonomic information, ranging from 82% to 100% and 94% to 99% nucleotide pairwise identity in 16S rRNA gene and fungi sequences, respectively. The criteria used for bacteria and fungi identification followed the same principles as indicated elsewhere [28,29,32].

### 2.7. Diversity Analysis of the Samples under Study 

Diversity analyses were performed through the calculation of four α-diversity indices, i.e., two richness indices (number of species and Chao1) and two diversity indices (Shannon index and Simpson index) [33].

### 2.8. Biotic and Abiotic Data Integration

For microbiota and bioenvironmental data integration, we performed a principal component analysis (PCA) using available information for all 20 Egyptian mongoose specimens. The biotic variables used were related to sex, age class, reproductive status, stomach content at the time of death, and different body measurements (body weight (BW), snout-tail length (STL), tail length (TL), head and body length (HBL), right hind leg length (RHLL), right hind foot length (RHFL), shoulder height (SH), neck perimeter (NP), head diameter (HD), head width (HW), spleen weight (SW), kidney weight (KW), solid fat index (SFI), and perivisceral fat index (PFI)). The body measurements were obtained as stated elsewhere [24]. The abiotic variables used were related to georeferenced location, land-use, climatic data, road net, river net, and population data. Both biotic and abiotic data available were coupled with the gut microbiota genera detected and a PCA was calculated, enabling the prediction of the most influential biotic and abiotic variables of the gut microbiota composition of the Egyptian mongoose. 

A matrix using 72 microbiota operational taxonomic units (OTUs), based on the presence/absence of every hierarchical bacterial level, was normalized using the standard score. The normalized matrix was used to perform an initial PCA. Scatter projection diagrams were obtained both for sex and age classes. Then, a dendrogram was made based on the normalized Euclidean distance derived from the projection matrix using UPGMA. The cophenetic correlation coefficient was calculated and a two-way Mantel test [34] was performed to measure the faithfulness of the dendrogram as compared with the pairwise distances of the original unmodeled matrix. Two other PCAs were made using a matrix with 26 biotic and 17 abiotic variables. These matrices were normalized using the standard score and PCAs were performed. Scatter projection diagrams were obtained for the clusters that originated from the previous microbiota dendrogram. The explanatory variables of each principal component (PC) were selected if the correlation coefficients between the variable and the PC were |0.5|. If a variable had this behavior with more than one PC, this variable was used as an explanatory variable for the PC with the higher correlation coefficient. These analyses were made using R software (Version 3.6.1, Vienna, Austria) and NTSYSpc software (version 2.20d; Exeter Software, Setauket, NY, USA).

### 2.9. Microbial Profiling Study

The culturomics data acquired in this study was compared with the data obtained from the same specimens by a microbial profiling approach [10]. Microbial profiling was performed as described elsewhere [10]. Briefly, the DNA was extracted from 500 mg of feces of each mongoose using the NZYSoil gDNA isolation kit (NZYTech, Lisbon, Portugal), followed by the amplification of the full-length 16S rRNA gene, and subsequently, the amplicons were sequenced on the PacBio SMRT RS-II platform (Pacific Biosciences, Menlo Park, CA, USA; commercially available at Eurofins Genomics, München, Germany). Data were preprocessed using the PacBio SMRT Analysis Portal (Pacific Biosciences, Menlo Park, CA, USA), generating single-molecule circular consensus sequences (CCS) that were analyzed using the EzBioCloud platform (Seoul, Korea).

### 2.10. Data Analysis

Considering culture assays, results from colony-forming units (CFU) counts are displayed as means of values of at least ten independent experiments with respective standard deviation. All variables were tested for normality using a D’Agostino–Pearson test (α = 0.05). Since samples did not pass the normality test, all subsequently used statistical testes were nonparametric. When comparing two conditions, a Mann–Whitney test (α = 0.05) was performed. When comparing multiple conditions, a Kruskal–Wallis test (α = 0.05) with a Dunn’s multiple comparison post-test was performed. When comparing multiple host communities, a Friedman test (α = 0.05) with a Dunn’s multiple comparison post-test was performed. When comparing relative abundances between methodological approaches, a Chi-square test (α = 0.05) was performed. All statistical analyses were performed using GraphPad Prism software (Version 7, La Jolla, CA, USA). 

## 3. Results

### 3.1. Evaluation of Microbial Burden across Sex and Age Classes

In order to examine the GI tract microbiota, 20 fecal samples of Egyptian mongoose specimens were used in a culture-dependent approach. We used two oxygenic conditions: aerobic (with (w/) O_2_) and anaerobic (without (w/o) O_2_) to incubate fecal samples in a rich medium, with (YCFA P) and without (YCFA) 0.1% of sodium taurocholate supplementation.

Microbial load in these four media/conditions (Supplementary Figure S2A) were compared; in male samples, an average of 2.6 × 10^9^ ± 3.0 × 10^9^ (YCFA w/ O_2_), 1.6 × 10^5^ ± 2.4 × 10^5^ (YCFA P w/ O_2_), 2.9 × 10^9^ ± 8.8 × 10^9^ (YCFA w/o O_2_), and 8.5 × 10^6^ ± 1.5 × 10^7^ CFU/g (YCFA P w/o O_2_). In female samples, we registered a mean of 3.0 × 10^9^ ± 2.6 × 10^9^ (YCFA w/ O_2_), 8.7 × 10^5^ ± 2.2 × 10^6^ (YCFA P w/ O_2_), 8.0 × 10^9^ ± 1.4 × 10^10^ (YCFA w/o O_2_), and 4.1 × 10^8^ ± 8.1 × 10^8^ CFU/g (YCFA P w/o O_2_). Regarding sex-related differences, female hosts had a significantly higher microbial load than males in YCFA w/o O_2_ (*p*-value = 0.0410, Mann–Whitney test, α = 0.05) and YCFA P w/o O_2_ (*p*-value = 0.0288, Mann–Whitney test, α = 0.05).

The microbial load in MacConkey medium had no significant differences across sexes, both for lactose non-fermenting (LNF) bacteria, lactose fermenting (LF) bacteria, and the sum of both types (Supplementary Figure S2B), with a mean of 2.1 × 10^9^ ± 4.7 × 10^9^, 1.8 × 10^9^ ± 4.7 × 10^9^, and 3.9 × 10^9^ ± 9.3 × 10^9^ CFU/g registered for male mongooses, respectively, and a mean of 8.1 × 10^9^ ± 1.0 × 10^10^, 3.9 × 10^9^ ± 6.2 × 10^9^, and 1.2 × 10^10^ ± 1.1 × 10^10^ CFU/g, respectively, in female mongooses.

In this study, we registered an association between the colony color and the morpho-physiological type of the isolate, in the ESBL chromogenic medium. The metallic blue colonies were associated with MT-II, light blue colonies associated with MT-VII, and pink colonies associated with MT-IX (presumptive *E. coli*). Colorless colonies were not associated with any specific MT. 

In ESBL w/o AS, in samples from male hosts, bacterial recovery was, on average, 3.1 × 10^12^ ± 2.5 × 10^12^ CFU/g; most colonies were colorless (3.1 × 10^12^ ± 2.5 × 10^12^ CFU/g) and MT-II (1.0 × 10^9^ ± 1.2 × 10^9^ CFU/g) and MT-VII (2.0 × 10^8^ ± 3.4 × 10^8^ CFU/g) were detected. The MT-IX colonies were not found (Supplementary Figure S2C). In samples from female hosts, an average of 3.6 × 10^12^ ± 1.2 × 10^12^ CFU/g was detected, which were mostly colorless colonies (3.6 × 10^12^ ± 1.2 × 10^12^ CFU/g), with MT-II (2.5 × 10^9^ ± 4.9 × 10^9^ CFU/g), MT-VII (5.8 × 10^8^ ± 1.4 × 10^9^ CFU/g), and MT-IX (9.0 × 10^7^ ± 2.4 × 10^8^ CFU/g) colonies also detected (number of hosts = 3) (Supplementary Figure S2C). Moreover, ESBL w/ AS presented an average bacterial load of 1.4 × 10^8^ ± 1.4 × 10^8^ CFU/g, with an even distribution between MT-II (1.0 × 10^8^ ± 1.4 × 10^8^ CFU/g) and MT-VII (3.6 × 10^7^ ± 6.8 × 10^7^ CFU/g) colonies, in male samples (Supplementary Figure S2D). In female samples, an average bacterial load of 1.9 × 10^7^ ± 2.5 × 10^7^ CFU/g, also with an even distribution between MT-II (1.6 × 10^8^ ± 2.4 × 10^8^ CFU/g) and MT-VII (3.1 × 10^7^ ± 8.8 × 10^7^ CFU/g) colonies, was registered. In both ESBL chromogenic media, there were no significant differences across sexes in the number of colonies from any color and morpho-physiological type (Supplementary Figure S2C,D). Bacterial growth in Brilliance ESBL medium was only registered in eight fecal samples, i.e., two fecal samples from male individuals and six from females, with a bacterial load average of 1.6 × 10^3^ ± 4.1 × 10^3^ and 6.8 × 10^3^ ± 1.1 × 10^4^ CFU/g, respectively. There were no significant differences noticed between the sexes.

The fungal community (mycobiota) present in the GI tract of mongoose was assessed through the use of the selective medium for fungi isolation, i.e., PDA medium supplemented with chloramphenicol (PDA w/CHLO) (Supplementary Figure S3). The average number of viable fungi found in fecal samples was 2.0 × 10^8^ ± 2.9 × 10^8^ and 1.3 × 10^7^ ± 2.9 × 10^7^ CFU/g, in males and females, respectively. Regarding yeast, no significant differences were shown across sexes (8.6 × 10^7^ ± 1.3 × 10^8^ and 1.3 × 10^7^ ± 2.9 × 10^7^ CFU/g, for males and females, respectively). However, comparing filamentous fungi (FF), significant differences (*p*-value = 0.0031, Mann–Whitney test, α = 0.05) between male and female samples were registered, with microbial loads in the former of approximately 1.2 × 10^8^ ± 1.9 × 10^8^ CFU/g, and no detection in the latter.

### 3.2. Evaluation of Morpho-Physiological Types across Sex and Age Classes

Following their purification, all isolates were characterized morpho-physiologically and grouped in several types (Supplementary Figure S1). On the basis of the morpho-physiology, the culturable gut microbiota was dominated by Gram-positive bacteria (76%) and rod-shaped bacteria (77%). The total gut microbiota was composed of several morpho-physiological types (n = 15), with MT-IX appearing in 100% of individuals, followed by MT-II in 90%, MT-VII in 70%, and MT-XI in 55% of individuals. Thus, the MT-II, MT-VII, MT-IX, and MT-XI types can be considered to be the core gut microbiota community, while the remaining morpho-physiological types encountered can be considered to be part of the intraspecific individual microbiota communities.

**Figure 1:**
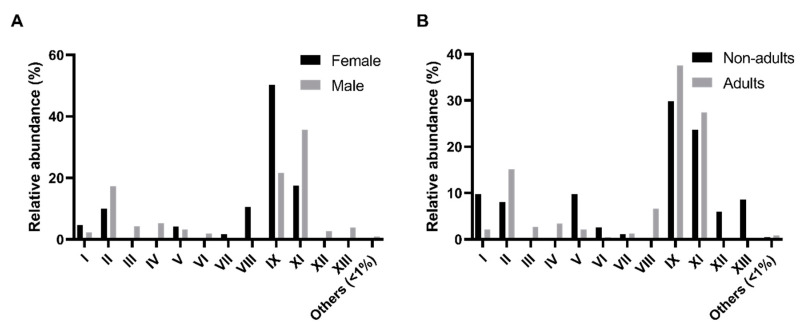
Comparison of the morpho-physiological types detected in the intestinal microbiota of the Egyptian mongoose specimens across sex and age classes. (**A**) Relative abundance in female and male individuals; (**B**) Relative abundance in nonadult and adult individuals.

The comparison of the relative abundance of these morpho-physiological types (MT) across sex and age classes was performed (Figure 1). A higher relative abundance of MT-IX isolates in female samples (*p*-value < 0.01, Friedman test, α = 0.05) was detected as compared with the male samples (Figure 1A). In males, MT-IX (100% of host individuals), MT-II (90%), MT-VII (80%), and MT-XI (60%) are the more representative types. The same happens in female samples which recorded 100% of MT-IX, 90% of MT-II, 60% of MT-VII, and 50% of MT-XI. Regarding age classes, there were no significant differences found (Figure 1B). The pattern of adults was similar with MT-IX (100%), MT-II (88%), MT-VII (69%), and MT-XI (63%) as the most represented. However, nonadults showed a slightly different pattern, with MT-II (100%), MT-IX (100%), MT-VII (75%), MT-XII (50%), and MT-XII (50%) as the core culturable types. 

### 3.3. Molecular Fingerprinting and Identification of Microbial Isolates

The molecular differentiation of isolates was based on RAPD fingerprints. The reproducibility of molecular fingerprints was estimated using the similarity average value for all pairs of duplicates, 97.2% ± 3.3% for M13, and 83.1% ± 9.6% for PH primer. A composite dendrogram based on molecular fingerprints from both primers was generated to accomplish the differentiation of bacterial isolates. A total of 122 clusters, of which 55 were single-member clusters, were generated using a cutoff value of 70% similarity. One isolate of each cluster was selected and identified through 16S rRNA gene sequencing. The isolates of each MT were grouped in individual dendrograms in order to diminish the entropy obtained from the massive diversity of isolates (Supplementary Figure S4). The identified bacterial isolates that were misplaced were regrouped into their correct MT dendrogram. After, one isolate of each newly formed unidentified cluster was selected for 16S rRNA gene sequencing.

**Figure 2:**
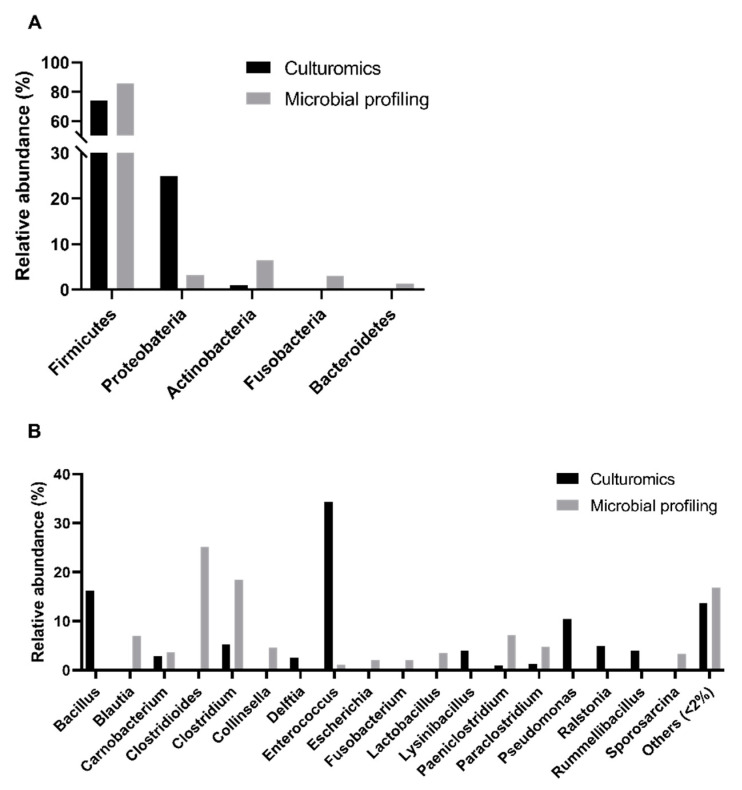
Comparison of the intestinal bacterial microbiota of the Egyptian mongoose between culturomics and microbial profiling [10] approaches. (**A**) Relative abundance at a phylum level; (**B**) Relative abundance at the genus level.

**Figure 3:**
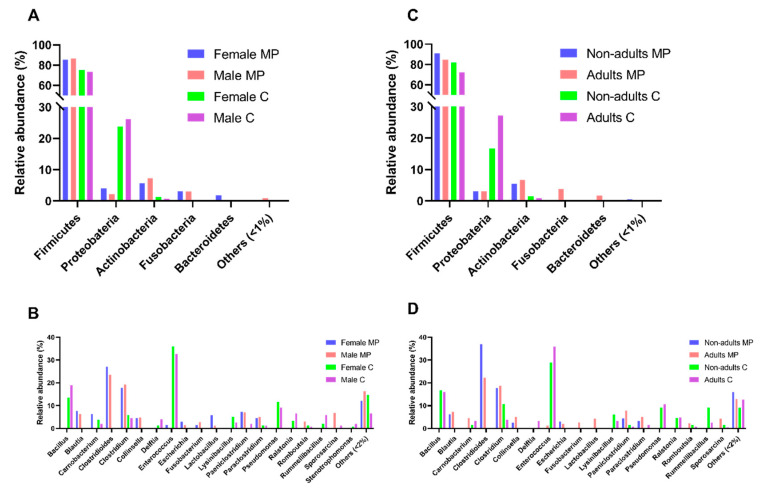
Comparison of the intestinal bacterial microbiota of Egyptian mongoose across sex and age classes, based on the datasets obtained through culturomics and microbial profiling [10] approaches. (**A**) Relative abundance on a phylum and (**B**) genus level for female and male individuals; (**C**) Relative abundance on a phylum and (**D**) genus level for nonadult and adult individuals.

Using a culturomics approach, we successfully identified bacterial isolates belonging to the following three phyla: *Firmicutes* (74% of bacterial isolates), *Proteobacteria* (25%), and *Actinobacteria* (1%) (Figure 2A, Supplementary Tables S1 and S2). However, in the previous study based on microbial profiling, five phyla with a relative abundance superior to 1% were detected, which enabled the disclosure of the presence of two other phyla: *Fusobacteria* (3%) and *Bacteroidetes* (1%). Additionally, when comparing the relative abundances of genera obtained through culturomics and microbial profiling, a significant difference was registered (*p*-value < 0.0001, Chi-square test, α = 0.05). In addition, there were no phyla exclusively detected in male, female, adult, or nonadult samples (Figure 3A,C) and, no significant differences were found across sex or age classes in culturomics data.

Overall, we found bacterial isolates from twenty genera (Figure 2B), with *Enterococcus* spp. (34%) and *Bacillus* spp. (16%) being the most abundant, while *Pseudomonas* spp. (10%), *Clostridium* spp. and *Ralstonia* spp. (5% each), *Lysinibacillus* spp. and *Rummeliibacillus* spp. (4% each) and *Carnobacterium* spp. and *Delftia* spp. (3% each) were less denoted. Some genera were rare including the following: *Paraclostridium* spp., *Stenotrophomonas* spp., *Paeniclostridium* spp., *Romboutsia* spp., *Sporosarcina* spp., *Staphylococcus* spp., *Paenibacillus* spp., *Pantoea* spp., *Cutibacterium* spp., *Psychrobacillus* spp., *Solibacillus* spp., and *Robinsoniella* spp. (1% each). We were not able to determine the genus of 6% of the cultured isolates submitted to 16S rRNA sequencing due to low homology with sequences deposited in public databases. However, in the previous microbial profiling analyses, *Clostridioides* spp. (25%), *Blautia* spp. (7%), *Collinsella* spp. (5%), *Lactobacillus* spp. (4%), *Escherichia* spp. and *Fusobacterium* spp. (2% each) were also reported. Contrarily, *Bacillus* spp., *Delftia* spp., *Lysinibacillus* spp., *Pseudomonas* spp., *Ralstonia* spp., and *Rummeliibacillus* spp. were only detected using the culturomics approach (this study). A statistically significant difference was registered between the relative abundances of genera detected through culturomics and microbial profiling (*p*-value < 0.0001, Chi-square test, α = 0.05).

The analysis of the presence/absence of each detected genus using both approaches was also performed at the fecal sample level. This analysis not only reinforces the notion that culturomics can provide additional information complementary to microbial profiling but also evidences the coherence between the cultivable and the total core gut microbiota of this mongoose population (Supplementary Figures S5 and S6).

The comparison of the male and female samples (Figure 3B) revealed that *Paeniclostridium* spp. and *Sporosarcina* spp. were exclusively cultured from the male samples, while *Psychrobacillus* spp. and *Staphylococcus* spp. were found exclusively in the female samples. The comparison of the adult and nonadult samples (Figure 3D) showed that *Delftia* spp., *Paraclostridium* spp., and *Rombinsoniella* spp. were only detected in the adult samples. There was no genus perceived to be unique to nonadults. No statistically significant differences were found across sex or age classes in the culturomics data.

Regarding the core bacterial microbiota, *Enterococcus* spp. was detected in 100% of the host individuals, *Bacillus* spp. in 90%, *Pseudomonas* spp. in 75%, *Ralstonia* spp. in 65%, and *Clostridium* spp. in 55%. Thus, these genera can be considered to be the core culturable bacterial microbiota of the Egyptian mongoose.

In addition to the bacterial isolates, we identified four fungi genera belonging to the phyla Basidiomycota (*Pseudozyma* spp. and *Naganishia albida*), Ascomycota (*Penicillium amaliae*), and Mucoromycota (*Mucor circinelloides*) (Supplementary Table S3).

### 3.4. Alpha Diversity Analysis

**Figure 4:**
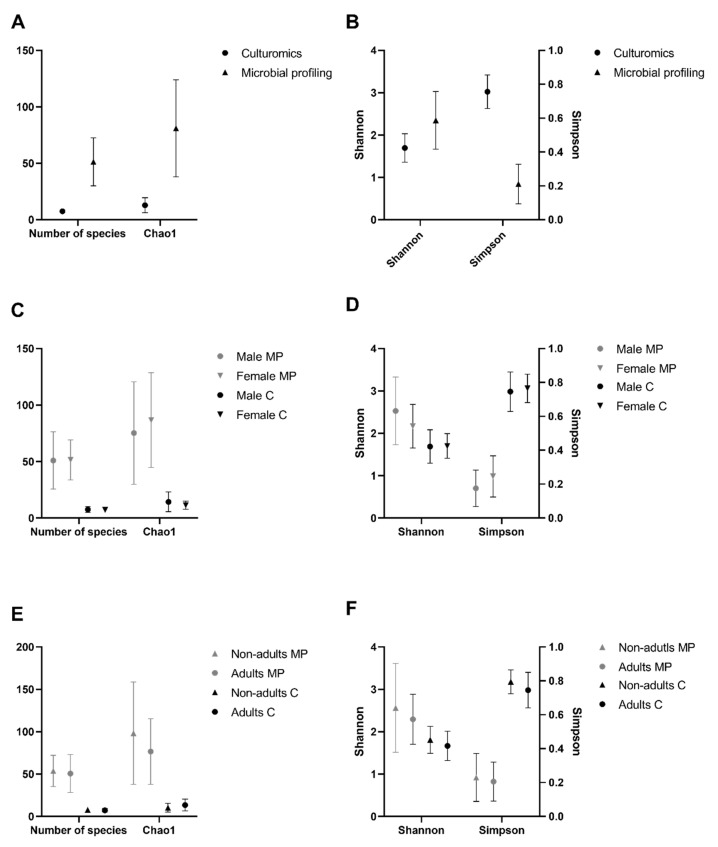
Comparison of the α-diversity indices across sex and age classes of Egyptian mongoose microbiota determined through culturomics and microbial profiling [10] approaches. (**A**) Richness indices (number of species and Chao1) and (**B**) diversity indices (Shannon and Simpson) of culturomics and microbial profiling approaches; (**C**) Richness indices (number of species and Chao1) and (**D**) diversity indices (Shannon and Simpson) of female and male individuals; (**E**) Richness indices (number of species and Chao1) and (**F**) diversity indices (Shannon and Simpson) of nonadult and adult individuals.

At the bacterial genus level, the α-diversity analysis was performed based on two richness indices (number of species and Chao1 index) and two diversity indices (Simpson index and Shannon index). Chao1 is a sensitive indicator of rare OTUs, with higher values indicating higher richness [35]. The Simpson and Shannon indices were used to assess the ratio between the number of individuals and the number of genera under analysis in each community, as they are the most commonly used in microbial community studies [36]. All indices, except the Simpson index, showed extremely significantly (*p*-value < 0.0001 in all cases, Friedman test, α = 0.05) higher values in the microbial profiling strategy than in the present culturomics study (Figure 4A,B). The Simpson index showed the opposite behavior, with extremely significantly (*p*-value < 0.0001, Friedman test, α = 0.05) higher values attained through culturomics. Additionally, diversity indices were used to compare sex and age classes. None of the indices showed any statistically significant differences, either across sex (Figure 4C,D) or age classes (Figure 4E,F).

### 3.5. Interaction between Culturable Microbiota and Bioenvironmental Features

**Figure 5:**
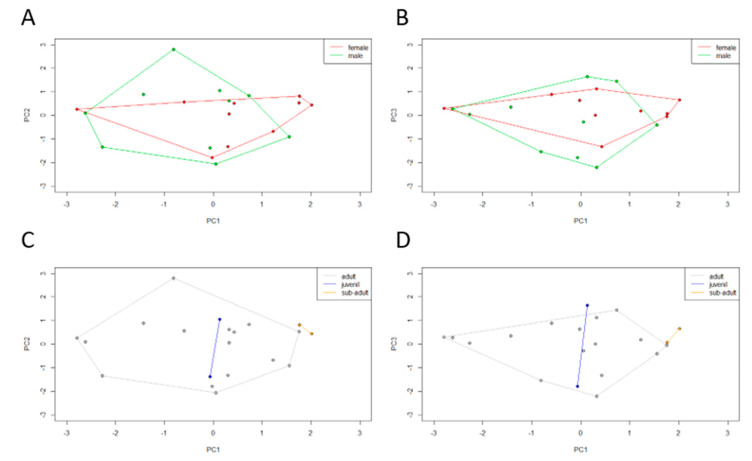
Two principal component (PC) plots depicting the dispersal area of microbiota data of each Egyptian mongoose specimen. The 20 specimens are projected in the (**A**,**C**) PC1 × PC2 and (**B**,**D**) PC1 × PC3 planes resulting from a principal component analysis performed on the Boolean matrix of the presence/absence of the different hierarchical bacterial levels. The vertices of each polygon correspond to the microbiota data observed for each specimen. Each polygon corresponds to (**A**,**B**) sex and (**C**,**D**) age class clustering.

In order to integrate culturable microbiota and bioenvironmental data, a PCA was performed using the microbiota composition of each individual (72 OTUs in global). The obtained PCA registered a cumulative variance of 42% (PC1 17%, PC2 13%, and PC3 12%). The dispersal areas of the microbiota data obtained for sex and age class were compared. Observing the dispersal area according to sex (Figure 5A,B), male and female clusters overlap almost completely. Observing the dispersal area according to age class (Figure 5C,D), a total overlap between juveniles and adults can be observed but subadult individuals form a differentiated cluster. Subadults tend to have a microbiota composed of *Clostridiaceae* and *Actinobacteria* phyla (represented in PC1). In the previous microbial profiling study, both sex and age class β-diversity analysis recorded no statistically significant differences, with an overlap between dispersal areas.

The dendrogram which originated from the normalized Euclidean distance, derived from the projection matrix using UPGMA, produced eight clusters, and five of them were single-member clusters when a cut-off value of 0.182 was used (Supplementary Figure S5). The two-way Mantel test showed a matrix correlation of 0.912. 

**Figure 6:**
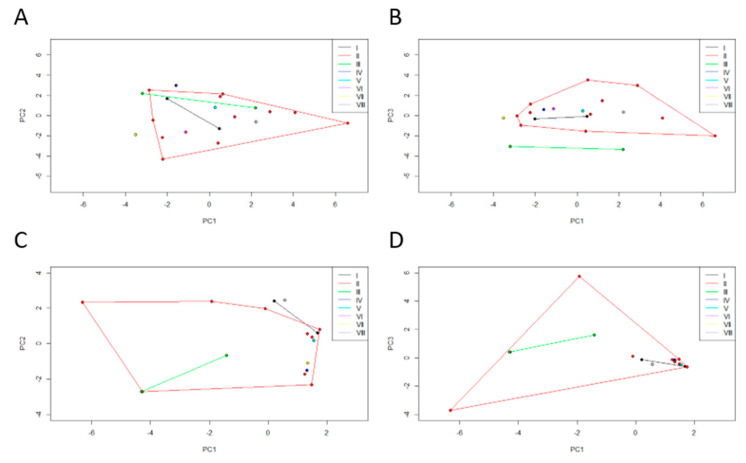
Two principal component (PC) plots depicting the dispersal area of (**A**,**B**) biotic and (**C**,**D**) abiotic data of each Egyptian mongoose specimen. The 20 specimens are projected in the (**A**,**C**) PC1 × PC2 and (**B**,**D**) PC1 × PC3 planes resulting from a principal component analysis performed on the data matrix. The vertices of each polygon correspond to the (**A**,**B**) biotic and (**C**,**D**) abiotic data observed for each specimen. Each polygon corresponds to the clusters originated from the microbiota data dendrogram.

Similarly, the dispersal areas of biotic (Figure 6A,B) and abiotic (Figure 6C,D) data obtained for the clusters originated from the previous microbiota dendrogram were compared. These PCA registered a cumulative variance of 55% (PC1 29%, PC2 14%, and PC3 11%) and 68% (PC1 34%, PC2, 18% and PC3 16%), respectively. We can see that cluster II is a very diverse group in terms of biological and environmental characteristics.

Regarding the biological characteristics, most of the clusters overlap completely, except cluster VII that is clearly distanced from the remaining clusters and clusters III and IV that only overlap partially. Cluster VII comprises the HI471 individual, who outlies due to high values of biometric features related to size, high values of biometric features related to weight, and stomach content at the time of death, is composed of a greater percentage of mammals and invertebrates. Cluster III is formed by the HI502 and HI516 individuals that show a higher reptile percentage in the stomach at the time of death. Cluster IV has the individual HI508, which has high values of biometric features related to size and low values of biometric features related to weight, but also a stomach content at the time of death is composed of a smaller percentage of mammals and invertebrates.

Regarding the environmental characteristics, most of the clusters overlap completely, except cluster VIII which only overlaps partially with the remaining clusters. Cluster VIII includes the individual HI388, whose land-use is mainly composed of agroforestry and mixed forest, and high river network; a high annual rainfall is characteristic of its habitat, while on the contrary, it has a low annual average temperature, annual thermal amplitude, and a reduced road network.

In the previous microbial profiling study, both biotic and abiotic factors influenced Egyptian mongoose gut microbiota, namely reptile and mammal stomach contents, as well as all of the biometric factors evaluated. Land-use, temperature, rainfall, river net, and road net were also shown to be influential predictors of microbiota composition.

## 4. Discussion

Gut microbiota is nowadays an important topic of investigation in biological and medical science due to its recognized importance in host biology and ecology. In this study, we generated extended baseline information on the culturable microbiome of mongoose, enabling the exploitation of microbial community differences across sex and age classes, the assessment of the influence exerted by the biological and environmental context of each host in its microbiota signature, and, given our previous work on the same specimens [11], the possibility to compare the datasets generated by culturomics and microbial profiling approaches. Culturomics is a recent technique based on an extensive culture-dependent approach using a large combination of different enrichment and culture conditions and high-throughput taxonomic identification of obtained isolates by mass spectrometry and/or DNA barcoding. This methodology has the ability to culture and thereafter characterize previously unculturable isolates, also enabling the detection of species never reported within microbiota communities, ultimately increasing the scientific knowledge on the biology and ecology of the studied host [11,12,13,14,37,38,39]. 

### 4.1. Overview of Results Offered by Culturomics

The microbial burden results showed that the aerobiota community was the most abundant, with an average of 3.3 × 10^12^ CFU/g of a fecal sample. Considering previous studies on carnivore mammals, as Egyptian mongoose, an average of 10^9^ CFU/g in grizzly and polar bears [3,40,41], dogs [42], and cats [43] fecal samples was reported. The anaerobiota community is normally the most studied community of gastrointestinal microbiota. In two previous studies, the YCFA medium was used as a medium that enabled the growth under anaerobic conditions of vast diversity and density of gut bacteria [26]. We report a mean of 5.5 × 10^9^ CFU/g of anaerobic bacteria, a value that is similar to a previous report on human fecal microbiota [44]. Sporobiota, this is, the community capable of producing spores, but also stress-resistant bacteria, showed to be less abundant, with an average of 5.2 × 10^5^ CFU/g under aerobiosis and 6.3 × 10^8^ CFU/g under anaerobiosis. These results are in agreement with previous sporobiota analyses in humans [26,45]. ESBLs are a very important and growing issue in human and animal health since they confer resistance to most β-lactam antibiotics, including penicillins, monobactams, as well as first-, second-, third-, and fourth-generation cephalosporins [46]. ESBL-producing and/or bacteria resistant to beta-lactams had an average abundance of 1.2 × 10^8^ CFU/g. Previous studies have reported the existence of fecal-oral cross-transmission of bacteria between the human population and wildlife [47]. Furthermore, the possibility of transmission of antibiotic-resistant bacteria within individuals from the *Herpestidae* family living in close contact with human areas has been hypothesized [48]. Refining the phenotype and genotype associated with ESBL-resistant bacteria isolated in this work (0.005%) could enable estimating how much beta-lactam-resistant bacteria circulate in wild Egyptian mongoose, in Portugal. Regarding sex-related differences, a higher microbial load of fecal samples from female hosts was quantified in YCFA w/o O_2_ and YCFA P w/o O_2_ media. Additionally, six of the eight samples that had culturable bacteria in Brilliance medium were from female hosts. 

The culturable microbiota community from the analyzed Egyptian mongoose gut specimens was dominated by Gram-positive bacteria (76%), mainly of the phylum *Firmicutes* (74%), with Bacilli isolates prevailing, in particular, *Enterococcus* spp. (34%) and *Bacillus* spp. (16%). Moreover, *Enterococcus* spp. was detected in 100% of the host individuals, *Bacillus* spp. in 90%, *Pseudomonas* spp. in 75%, *Ralstonia* spp. in 65%, and *Clostridium* spp. in 55%. Thus, these genera can be considered the core culturable bacterial microbiota of Egyptian mongoose. *Paeniclostridium* spp. and *Sporosarcina* spp. were exclusively detected in male samples, *Psychrobacillus* spp. and *Staphylococcus* spp. in female samples, and *Delftia* spp., *Paraclostridium* spp., and *Rombinsoniella* spp. in adult samples. No genus was perceived as unique to nonadults. All the uniquely found genera registered a relative abundance lower than 5%, therefore, differences can be explained by intraspecific variation within individual hosts and not likely by sex or age class features. 

In the mammals’ GI tract, fungi are found to be present in smaller amounts as compared with bacteria but they are believed to be of extreme importance in host biology. The core gut mycobiota from the Egyptian mongoose specimens registered an average of 1.1 × 10^8^ CFU/g, a higher amount as compared with previous studies in humans and mice [49,50,51]. A statistically significant higher burden of filamentous fungi was found across the male samples. We only detected four fungi genera, all previously described as gut commensals of mammals. *Penicillium* and *Naganishia* genera are commensal fungi previously found in Pygmy Loris [52], Yunnan snub-nosed monkeys [53], and humans [54]. *Penicillium* spp. form a lot of microscopic spores that are frequently found in air and soil and can easily be inhaled or ingested during the feeding process, and are able to grow on the GI tract of mammals [54]. *Pseudozyma* members are normally found associated with plants and are also part of the normal gut microbiota of Giant pandas [55]. *Mucor* spp. was previously found in the GI tract of human individuals, but its function on the gut microbiota is still unknown [54].

### 4.2. Level of Information Attained with Culturomics vs. Microbial Profiling

In agreement with microbial profiling, culturomics showed that the core gut cultivable microbiota of mongoose is dominated by *Firmicutes* and, as the previous approach, is able to distinguish sex- and age class-specific genera. Comparing the microbial taxa with a relative abundance equal or superior to 1% obtained through culturomics in the present study and, previously, by microbial profiling, two unique phyla stand out from the culture-independent approach, i.e., *Fusobacteria* (3%) and *Bacteroidetes* (1%). Additionally, microbial profiling enabled the detection of the following six other genera: *Clostridioides* spp. (25%), *Blautia* spp. (7%), *Collinsella* spp. (5%), *Lactobacillus* spp. (4%), and *Escherichia* spp. and *Fusobacterium* spp. (2% each). Most of the taxa exclusively detected using a microbial profiling approach were anaerobic, non-spore-forming bacteria. Strict anaerobic growth conditions are hard to maintain, which could lead to a quick loss of cell viability, particularly in non-spore-forming bacteria, resulting in the non-cultivability of those bacteria. In contrast, *Bacillus* spp., *Delftia* spp., *Lysinibacillus* spp., *Pseudomonas* spp., *Ralstonia* spp., and *Rummeliibacillus* spp. were only detected using the culturomics approach adopted in this study. Those genera belonging to the *Bacillales* order are spore-forming bacteria. Pasteurization and medium supplementation with sodium taurocholate promoted their cultivability. The remaining genera belong to the *Proteobacteria* phylum, an easily cultivable group of bacteria. *Bacillus*, *Lysinibacillus*, and *Rummeliibacillus* genera are spore-forming bacteria and *Deftia*, *Pseudomonas*, and *Ralstonia* genera are Gram-negative bacteria, very well known to possess an outer membrane, both characteristics that make these genera less susceptible to lysis during the DNA extraction procedure, which could lead to a representability bias (lower representation) in the microbial profiling study.

Regarding the biological functions attributed to the genera uniquely found using culturomics, those belonging to the *Bacillales* order are taken as responsible for protein degradation, gut homeostasis, host immunity development, and extended host lifespan, influencing host gene regulation of immune factors and cell proliferation, as well as the availability of key vitamins and cofactors [56,57]. Gammaproteobacteria, namely *Pseudomonas* spp., is one of the most common bacterial groups in the mammal GI tract, which is responsible for the breakdown and fermentation of complex sugars and the production of vitamins [56]. Regarding Betaproteobacteria, the *Ralstonia* spp. has been reported as being responsible for the increase of glucose intolerance in obese humans and mice [58] and *Delftia* spp. has been commonly found in the gastrointestinal microbiota of insects and fishes [59,60].

Comparisons of α-diversity at the bacterial genus level were performed using the results from culture-independent and culture-dependent approaches. However, interpretation of results requires caution as it should be kept in mind that there was a bottleneck in the identification of taxa from culturomics related with picking morphologically different colonies and the subsequent methodological steps and criteria to select and identify the isolates (please see methods Section 2.3, Section 2.4 and Section 2.5). These different stages that led to a progressive reduction in the panel of isolates to a final set of 500 can lead to a bias towards the detection of new genera, and consequently, have an implication on the α-diversity analysis, since those indices highly depend on the number of detected taxa and also on their relative abundance. Circumvention of these limitations would be completed if all culturable isolates had been subject to molecular identification, which would mean 16S rRNA, 26SrRNA and ITS sequencing of thousands and thousands of isolates. Thus, the direct comparison between culturomics and microbial profiling approaches needs to be made carefully. 

Despite these constrains, richness indices were concordant between culture-dependent and culture-independent approaches, registering statistically significant higher values using the microbial profiling approach over the culturomics dataset. However, the diversity indices behaved differently, with the Shannon index evidencing higher values for the microbial profiling data and the Simpson index yielding higher values for the dataset attained through culturomics. These contrasting results were due to a differential influence of dominant and rare taxa on those indices. The Shannon index is mainly influenced by species richness and rare taxa, being very sensitive to even very small diversity changes [61]. However, the Simpson index is more influenced by evenness and dominant taxa, not being affected by the less abundant [61]. Therefore, the recorded differences between Shannon and Simpson indices demonstrate that the microbial profiling approach is able to discover more taxa, but many correspond to groups of bacteria with very low relative abundance (less than 1%). Those taxa are enclosed in the great majority of microbial profiling studies but normally they are not under focus due to the enormous amount of generated data by direct sequencing methodologies in which more abundant taxa stand out; but also due to the low reliability of results when it comes to the less represented taxa. These results highlight the limitation of culturomics that detects fewer taxa. Nevertheless, these could represent the dominant cultivable population and possibly the most metabolically active or viable.

Regarding β-diversity analyses, there were no differences of culturable taxa found across sexes, confirming previous results from microbial profiling. The beta diversity analyses of culturable microbiota showed similarities between adults and juveniles. This similarity could be due to the higher proximity and interaction between these two age class groups since mongoose has a social behavior that implies protection and feeding of the cubs, scent marking, and social latrines, which increases diet similarity, and fecal-oral transmission of microbiota [62]. This type of social behavior could ease the host-to-host transmission of microbiota, as it was already observed in captive and wild animals, such as mice, birds, and humans [4]. However, there were no differences among age classes found using the microbial profiling approach. A clear association between the culturable microbiota of Egyptian mongoose and the bioenvironmental characteristics of each sampled animal was not demonstrated. However, in the microbial profiling approach, several abiotic and biotic factors were detected as relevant for Egyptian mongoose bacterial gut microbiota. 

The discrepancies in the inferences resulting from the β-diversity analyses and the assessment of the influence of biotic and abiotic factors between the two approaches could be related to a lower potential of culturomics as compared with microbial profiling, to detect viable but non-culturable bacteria or metabolically injured microorganisms. Thus, sequence-based methods have demonstrated their superior ability to detect more taxa, enhancing the discovery of a wide spectrum of gut microbiota changes across different individuals, and therefore the ability to assess at a fine-scale the influence exerted by several abiotic and biotic factors on gut composition and function.

## 5. Conclusions

The use of two complementary methodological approaches to study microbiota, i.e., culturomics and microbial profiling (culture-independent), enabled the following: (1) complementary detection of taxa, with emphasis on the ability of each approach to discover novel specific bacterial groups; (2) comparison of the resolution provided by each approach to evaluate sex- and age class-related differences; (3) assessment of the power of each approach to discriminate the influential effects of biotic- and abiotic- factors; and (4) exposure of the advantages and limitations of each strategy.

The culturomics approach was essential to achieve the following: (1) add information on the cultivability of the Egyptian mongoose gut microbiota according to bacterial functional groups (aerobiota, anaerobiota, sporobiota, and mycobiota), both in terms of microbial burden and operational taxonomical units; (2) increase knowledge of the differences between the microbial community composition of different age classes, namely between adults/juveniles and subadults; (3) offer the possibility of future work focusing on the phenotypic and genotypic characteristics of the recovered isolates, namely regarding virulence and pathogenic features and, also, antimicrobial resistance determinants, which could provide clues on the exposure of mongoose to anthropogenic effects as previously suggested [63] and expand knowledge of this species’ bio-ecology.

Several limitations could be highlighted with respect to the culturomics approach, namely the probable underestimation of microbial abundance and diversity due to difficult cultivability of fastidious microorganisms and the intensive and time-consuming characteristics of the approach that inevitably lead to lower throughput and analyses biases, which should be considered when choosing this approach over alternative methods.

## Supplementary Materials

The following materials are available online at https://www.mdpi.com/2076-2607/8/6/808/s1, Figure S1: Flowchart used for morpho-physiological differentiation purposes, Figure S2: Microbial load in selective and non-selective media, Figure S3: Microbial load in PDA medium with chloramphenicol, Figure S4: Bacterial isolates identification and differentiation by a hierarchical numerical analysis, Figure S5: Genera distribution detected by each methodological approach per fecal sample, Figure S6: Genera distribution per fecal sample, Figure S7: Dendrogram of microbiota relationships resulting from the PCA, Table S1: Information on the 16S rDNA, Table S2: Relative abundances, at the bacterial genus level, of each taxon in culturomics and microbial profiling approaches, Table S3: Information on the ITS nucleotide sequences of a selected group of fungi isolates.
