# Supplementary material for: Crosstalk Between Culturomics and Microbial Profiling of Egyptian Mongoose (Herpestes ichneumon) Gut Microbiome
Source: Microorganisms. 2020 May 27;8(6):808. doi: 10.3390/microorganisms8060808 (PMC7355707; doi:10.3390/microorganisms8060808)
Supplement: Supplementary file 1 [file microorganisms-08-00808-s001.pdf]

Supplementary Table 1 – Information on the 16S rDNA nucleotide sequences of a selected group of bacterial isolates.

**Isolate**	**Animal**	**Phenotypic Identification[1]**	**Nucleotide Sequence Length**	**Closest Reference Sequence Match**	**Query cover**	**E-value**	**Nucleotide Sequence Identity**	**Accession Number**	**Assigned Phylotype***
8	462	IX	1087	Pseudomonas plecoglossicida strain NBRC 103162 16S ribosomal RNA gene, partial sequence	99%	0.0	99%	NR_114226.1	*Pseudomonas*
9	467	VII	638	[Clostridium] bifermentans strain JCM 1386 16S ribosomal RNA gene, partial sequence	97%	0.0	99%	NR_113323.1	*Paraclostridium*
11	466	II	246	Enterococcus faecium strain NBRC 100486 16S ribosomal RNA gene, partial sequence	95%	0.0	99%	NR_113904.1	*Enterococcus*
22	462	I	1136	Staphylococcus saprophyticus subsp. bovis strain GTC 843 16S ribosomal RNA gene, partial sequence	100%	0.0	99%	NR_041324.1	*Staphylococcus*
29	462	V	582	Shigella sonnei strain CECT 4887 16S ribosomal RNA gene, partial sequence	99%	0.0	99%	NR_104826.1	*Enterobacteriaceae*
43	467	IX	1049	Clostridium baratii strain IP 2227 16S ribosomal RNA gene, complete sequence	100%	0.0	99%	NR_029229.1	*Clostridium baratii*

54	383	IX	710	[Eubacterium] tenue strain ATCC 25553 16S ribosomal RNA gene, partial sequence	99%	0.0	97%	NR_115794.1	*Paeniclostridium*
72	467	VII	1036	[Clostridium] sordellii strain JCM 3814 16S ribosomal RNA gene, partial sequence	100%	0.0	98%	NR_113140.1	*Peptostreptococcaceae*
79	399	VII	1095	Romboutsia lituseburensis strain ATCC 25759 16S ribosomal RNA gene, partial sequence	99%	0.0	98%	NR_118728.1	*Romboutsia*
84	462	VII	455	[Clostridium] bifermentans strain JCM 1386 16S ribosomal RNA gene, partial sequence	100%	0.0	99%	NR_113323.1	*Paraclostridium bifermentans*
91	501	IX	400	Bacillus anthracis strain SBS1 16S ribosomal RNA gene, partial sequence	87%	2,00E-149	99%	NR_118536.1	*Bacillus anthracis*
101	519	VII	617	[Clostridium] bifermentans strain JCM 1386 16S ribosomal RNA gene, partial sequence	100%	0.0	99%	NR_113323.1	*Paraclostridium bifermentans*
104	519	X	1001	Clostridium perfringens strain ATCC 13124 16S ribosomal RNA gene, complete sequence	99%	0.0	99%	NR_121697.1	*Clostridium perfringens*
116	636	II	600	Enterococcus faecalis strain NBRC 100480	100%	0.0	94%	NR_113901.1	*Enterococcaceae*

				16S ribosomal RNA gene, partial sequence					
117	636	VII	955	Clostridium tertium strain JCM 6289 16S ribosomal RNA gene, partial sequence	100%	0.0	99%	NR_113325.1	*Clostridium tertium*
131	516	VII	1018	[Clostridium] bifermentans strain JCM 1386 16S ribosomal RNA gene, partial sequence	99%	0.0	99%	NR_113323.1	*Paraclostridium bifermentans*
137	516	III	719	Stenotrophomonas maltophilia strain ATCC 13637 16S ribosomal RNA gene, partial sequence	100%	0.0	98%	NR_112030.1	*Stenotrophomonas*
147	501	VII	958	Bacillus thuringiensis strain NBRC 101235 16S ribosomal RNA gene, partial sequence	99%	0.0	98%	NR_112780.1	*Bacillus*
153	516	II	985	Enterococcus hirae strain ATCC 9790 16S ribosomal RNA gene, complete sequence	99%	0.0	99%	NR_075022.1	*Enterococcus*
170	396	IX	1062	Pseudomonas plecoglossicida strain NBRC 103162 16S ribosomal RNA gene, partial sequence	100%	0.0	99%	NR_114226.1	*Pseudomonas*
184	462	II	629	Pseudomonas alcaligenes strain IAM 12411 16S ribosomal	52%	3,00E-85	82%	NR_043419.1	*Pseudomonadales*

				RNA gene, complete sequence					
205	467	III	675	Hafnia alvei strain JCM 1666 16S ribosomal RNA gene, partial sequence	99%	0.0	99%	NR_112985.1	*Hafniaceae*
215	466	IX	966	Pseudomonas nitritireducens strain WZBFD3-5A2 16S ribosomal RNA, partial sequence	99%	0.0	99%	NR_133020.1	*Pseudomonas*
221	396	IX	400	Pseudomonas nitroreducens strain IAM1439 16S ribosomal RNA gene, complete sequence	96%	0.0	98%	NR_115611.1	*Pseudomonas*
224	467	IV	1046	Pseudomonas nitritireducens strain WZBFD3-5A2 16S ribosomal RNA, partial sequence	99%	0.0	99%	NR_133020.1	*Pseudomonas*
225	467	III	566	Ralstonia pickettii strain NBRC 102503 16S ribosomal RNA gene, partial sequence	100%	0.0	98%	NR_114126.1	*Ralstonia*
235	466	II	992	Enterococcus faecalis strain NBRC 100480 16S ribosomal RNA gene, partial sequence	99%	0.0	99%	NR_113901.1	*Enterococcus faecalis*
246	466	IX	474	Lysinibacillus sphaericus strain NBRC 15095 16S ribosomal	100%	0.0	98%	NR_112627.1	*Lysinibacillus*

				RNA gene, partial sequence					
248	462	IX	1014	Shigella boydii strain P288 16S ribosomal RNA gene, partial sequence	100%	0.0	99%	NR_104901.1	*Enterobacteriaceae*
260	462	VII	1032	Delftia lacustris strain 332 16S ribosomal RNA gene, partial sequence	99%	0.0	99%	NR_116495.1	*Delftia*
261	462	IV	773	Ralstonia pickettii strain NBRC 102503 16S ribosomal RNA gene, partial sequence	100%	0.0	99%	NR_114126.1	*Ralstonia pickettii*
271	396	IX	901	Pseudomonas nitritireducens strain WZBFD3-5A2 16S ribosomal RNA, partial sequence	97%	0.0	97%	NR_133020.1	*Pseudomonas*
293	505	IX	1043	Pseudomonas plecoglossicida strain NBRC 103162 16S ribosomal RNA gene, partial sequence	99%	0.0	99%	NR_114226.1	*Pseudomonas*
302	396	IX	1070	Bacillus anthracis strain ATCC 14578 16S ribosomal RNA gene, partial sequence	100%	0.0	98%	NR_041248.1	*Bacillus*
320	501	VII	1102	Bacillus thuringiensis strain NBRC 101235 16S ribosomal RNA gene, partial sequence	98%	0.0	99%	NR_112780.1	*Bacillus*

325	501	VII	656	Bacillus marcorestinctum strain LQQ 16S ribosomal RNA gene, partial sequence	93%	0.0	92%	NR_117414.1	*Bacillaceae*
338	519	VII	965	Bacillus cereus ATCC 14579 16S ribosomal RNA (rrnA) gene, complete sequence	98%	0.0	99%	NR_074540.1	*Bacillus*
340	516	VII	937	Bacillus toyonensis strain BCT-7112 16S ribosomal RNA gene, complete sequence	100%	0.0	99%	NR_121761.1	*Bacillus*
344	516	II	248	Enterococcus faecalis strain NBRC 100480 16S ribosomal RNA gene, partial sequence	96%	2E-121	99%	NR_113901.1	*Enterococcus faecalis*
349	462	VII	1052	Shigella boydii strain P288 16S ribosomal RNA gene, partial sequence	99%	0.0	99%	NR_104901.1	*Enterobacteriaceae*
366	399	VII	1073	Enterococcus faecium strain DSM 20477 16S ribosomal RNA gene, complete sequence	99%	0.0	99%	NR_114742.1	*Enterococcus*
388	388	II	1050	Enterococcus hirae strain ATCC 9790 16S ribosomal RNA gene, complete sequence	100%	0.0	99%	NR_075022.1	*Enterococcus*
390	509	XI	303	Bacillus anthracis strain SBS1 16S ribosomal	94%	3,00E-116	99%	NR_118536.1	*Bacillus anthracis*

				RNA gene, partial sequence					
456	516	OTHER	676	Rummeliibacillus stabekisii strain NBRC 104870 16S ribosomal RNA gene, partial sequence	98%	0.0	99%	NR_114270.1	*Rummeliibacillus stabekisii*
462	516	VIII	951	Rummeliibacillus stabekisii strain NBRC 104870 16S ribosomal RNA gene, partial sequence	100%	0.0	99%	NR_114270.1	*Rummeliibacillus stabekisii*
477	501	VIBRIUM	606	Ralstonia pickettii strain NBRC 102503 16S ribosomal RNA gene, partial sequence	100%	0.0	99%	NR_114126.1	*Ralstonia pickettii*
492	504	II	1094	Enterococcus faecium strain DSM 20477 16S ribosomal RNA gene, complete sequence	99%	0.0	99%	NR_114742.1	*Enterococcus*
499	504	OTHER	1027	Enterococcus faecium strain DSM 20477 16S ribosomal RNA gene, complete sequence	100%	0.0	99%	NR_114742.1	*Enterococcus*
505	508	II	950	Pseudomonas nitritireducens strain WZBFD3-5A2 16S ribosomal RNA, partial sequence	97%	0.0	97%	NR_133020.1	*Pseudomonas*
506	508	XI	1110	Carnobacterium gallinarum strain DSM 4847 16S ribosomal	99%	0.0	98%	NR_042093.1	*Carnobacterium*

				RNA gene, complete sequence					
509	508	IX	1150	Lysinibacillus fusiformis strain NBRC15717 16S ribosomal RNA gene, partial sequence	100%	0.0	98%	NR_112569.1	*Lysinibacillus*
531	505	V	122	Lysinibacillus alkaliphilus strain OMN17 16S ribosomal RNA, partial sequence	71%	3,00E-37	98%	NR_136779.1	*Lysinibacillus*
541	636	II	491	Enterococcus hirae strain LMG 6399 16S ribosomal RNA gene, complete sequence	99%	0.0	99%	NR_114783.2	*Enterococcus*
559	388	X	1004	Lysinibacillus fusiformis strain NBRC15717 16S ribosomal RNA gene, partial sequence	100%	0.0	99%	NR_112569.1	*Lysinibacillus fusiformis*
589	504	VII	401	Bacillus toyonensis strain BCT-7112 16S ribosomal RNA gene, complete sequence	99%	0.0	98%	NR_121761.1	*Bacillus*
596	508	VIII	500	Bacillus pumilus strain NRRL NRS-272 16S ribosomal RNA gene, partial sequence	100%	0.0	98%	NR_116191.1	*Bacillus*
611	466	II	974	Enterococcus faecium strain NBRC 100486 16S ribosomal RNA gene, partial sequence	100%	0.0	98%	NR_113904.1	*Enterococcus*

626	467	II	401	Enterococcus faecium strain DSM 20477 16S ribosomal RNA gene, complete sequence	100%	0.0	100%	NR_114742.1	*Enterococcus faecium*
627	467	OTHER	1047	Delftia lacustris strain 332 16S ribosomal RNA gene, partial sequence	100%	0.0	99%	NR_116495.1	*Delftia lacustris*
628	467	IX	856	Escherichia fergusonii strain ATCC 35469 16S ribosomal RNA gene, complete sequence	100%	0.0	99%	NR_074902.1	*Enterobacteriaceae*
629	467	OTHER	851	Pseudomonas nitroreducens strain NBRC 12694 16S ribosomal RNA gene, partial sequence	96%	0.0	98%	NR_113601.1	*Pseudomonas*
630	467	OTHER	410	Stenotrophomonas maltophilia strain NBRC 14161 16S ribosomal RNA gene, partial sequence	60%	5,00E-110	95%	NR_113648.1	*Xanthomonadaceae*
640	462	II	912	Enterococcus durans strain JCM 8725 16S ribosomal RNA gene, partial sequence	100%	0.0	98%	NR_113257.1	*Enterococcus*
665	383	IX	980	Pseudomonas nitritireducens strain WZBFD3-5A2 16S ribosomal RNA, partial sequence	99%	0.0	98%	NR_133020.1	*Pseudomonas*
670	467	IX	747	Delftia tsuruhatensis strain NBRC 16741 16S	100%	0.0	99%	NR_113870.1	*Delftia*

				ribosomal RNA gene, partial sequence					
680	502	V	224	Bacillus thuringiensis strain NBRC 101235 16S ribosomal RNA gene, partial sequence	99%	2,00E-111	99%	NR_112780.1	*Bacillus*
687	502	OTHER	408	Stenotrophomonas maltophilia strain ATCC 19861 16S ribosomal RNA gene, complete sequence	99%	0.0	96%	NR_040804.1	*Stenotrophomonas*
690	502	OTHER	584	Stenotrophomonas maltophilia strain ATCC 13637 16S ribosomal RNA gene, partial sequence	100%	0.0	97%	NR_112030.1	*Stenotrophomonas*
691	502	OTHER	740	Stenotrophomonas maltophilia strain ATCC 13637 16S ribosomal RNA gene, partial sequence	99%	0.0	98%	NR_112030.1	*Stenotrophomonas*
726	471	II	1133	Enterococcus hirae strain ATCC 9790 16S ribosomal RNA gene, complete sequence	99%	0.0	99%	NR_075022.1	*Enterococcus*
734	471	VII	1039	Bacillus toyonensis strain BCT-7112 16S ribosomal RNA gene, complete sequence	98%	0.0	99%	NR_121761.1	*Bacillus*
738	471	IV	1100	Ralstonia insidiosa strain AU2944 16S	100%	0.0	99%	NR_025242.1	*Ralstonia insidiosa*

				ribosomal RNA gene, partial sequence					
746	471	V	236	Rummeliibacillus stabekisii strain NBRC 104870 16S ribosomal RNA gene, partial sequence	95%	1E-114	99%	NR_114270.1	*Rummeliibacillus stabekisii*
747	471	V	400	Sporosarcina soli strain I80 16S ribosomal RNA gene, partial sequence	95%	0.0	98%	NR_043527.1	*Sporosarcina*
756	460	XI	678	Ralstonia pickettii strain NBRC 102503 16S ribosomal RNA gene, partial sequence	100%	0.0	99%	NR_114126.1	*Ralstonia pickettii*
794	399	IX	449	Staphylococcus saprophyticus strain ATCC 15305 16S ribosomal RNA gene, partial sequence	100%	0.0	99%	NR_115607.1	*Staphylococcus*
802	463	XI	668	Ralstonia insidiosa strain AU2944 16S ribosomal RNA gene, partial sequence	99%	0.0	99%	NR_025242.1	*Ralstonia insidiosa*
805	463	VII	360	Bacillus anthracis strain ATCC 14578 16S ribosomal RNA gene, partial sequence	97%	0.0	100%	NR_041248.1	*Bacillus anthracis*
837	508	V	752	Bacillus cereus ATCC 14579 16S ribosomal RNA (rrnA) gene, complete sequence	100%	0.0	99%	NR_074540.1	*Bacillus*

852	675	II	477	Pseudomonas nitritireducens strain WZBFD3-5A2 16S ribosomal RNA, partial sequence	84%	2E-160	92%	NR_133020.1	*Pseudomonadaceae*
864	471	VIII	968	Lysinibacillus sphaericus strain NBRC 15095 16S ribosomal RNA gene, partial sequence	99%	0.0	98%	NR_112627.1	*Lysinibacillus*
869	471	II	963	Enterococcus mundtii strain NBRC 100490 16S ribosomal RNA gene, partial sequence	100%	0.0	99%	NR_113906.1	*Enterococcus mundtii*
879	505	II	571	Lysinibacillus sphaericus strain NBRC 15095 16S ribosomal RNA gene, partial sequence	100%	0.0	98%	NR_112627.1	*Lysinibacillus*
889	504	VIII	1145	Lysinibacillus sphaericus strain NBRC 15095 16S ribosomal RNA gene, partial sequence	100%	0.0	98%	NR_112627.1	*Lysinibacillus*
915	516	II	252	Enterococcus faecalis strain NBRC 100480 16S ribosomal RNA gene, partial sequence	94%	1E-113	98%	NR_113901.1	*Enterococcus*
919	463	VI	220	Rummeliibacillus stabekisii strain NBRC 104870 16S ribosomal RNA gene, partial sequence	98%	3,00E-110	99%	NR_114270.1	*Rummeliibacillus stabekisii*

923	463	VIII	400	Lysinibacillus macroides strain LMG 18474 16S ribosomal RNA gene, partial sequence	72%	0%	97%	NR_114920.1	*Lysinibacillus*
928	502	VI	139	Geobacillus thermoglucosidasius strain R-35637 16S ribosomal RNA gene, partial sequence	61%	3,00E-28	92%	NR_116983.1	*Bacillaceae*
942	466	VI	447	Bacillus cereus ATCC 14579 16S ribosomal RNA (rrnA) gene, complete sequence	100%	0.0	100%	NR_074540.1	*Bacillus*
952	502	VI	412	Romboutsia lituseburensis strain ATCC 25759 16S ribosomal RNA gene, partial sequence	100%	0.0	98%	NR_118728.1	*Romboutsia*
953G	502	OTHER	801	Pseudomonas nitroreducens strain NBRC 12694 16S ribosomal RNA gene, partial sequence	99%	0.0	98%	NR_113601.1	*Pseudomonas*
953P	502	OTHER	650	Sporosarcina newyorkensis strain 6062 16S ribosomal RNA gene, partial sequence	100%	0.0	99%	NR_117567.1	*Sporosarcina newyorkensis*
956	502	V	312	Bacillus subtilis subsp. inaquosorum strain BGSC 3A28 16S	97%	4,00E-160	100%	NR_104873.1	*Bacillus*

				ribosomal RNA gene, partial sequence					
963	504	VI	396	Rummeliibacillus stabekisii strain NBRC 104870 16S ribosomal RNA gene, partial sequence	98%	0.0	99%	NR_114270.1	*Rummeliibacillus stabekisii*
966	463	II	630	Pseudomonas taiwanensis strain BCRC 17751 16S ribosomal RNA gene, partial sequence	61%	6,00E-106	85%	NR_116172.1	*Pseudomonadales*
978	471	VI	677	Bacillus toyonensis strain BCT-7112 16S ribosomal RNA gene, complete sequence	99%	0.0	99%	NR_121761.1	*Bacillus*
988	460	VIII	531	Pseudomonas nitroreducens strain NBRC 12694 16S ribosomal RNA gene, partial sequence	99%	0.0	98%	NR_113601.1	*Pseudomonas*
998	516	IX	637	Solibacillus silvestris strain HR3-23 16S ribosomal RNA gene, partial sequence	99%	0.0	99%	NR_028865.1	*Solibacillus*
1001	388	VIII	448	Lysinibacillus xylanilyticus strain XDB9 16S ribosomal RNA gene, partial sequence	93%	0.0	98%	NR_116698.1	*Lysinibacillus*
1004	388	VI	903	Psychrobacillus soli strain NHI-2 16S	89%	0.0	99%	NR_137244.1	*Psychrobacillus soli*

				ribosomal RNA, partial sequence					
1020	383	VII	86	Uncultured bacterium partial 16S rRNA gene, clone 1611_28_123	33%	3,00E-04	100%	LT173941.1	*Enterobacteriaceae*
1023	383	VII	400	Delftia tsuruhatensis strain NBRC 16741 16S ribosomal RNA gene, partial sequence	99%	0.0	99%	NR_113870.1	*Delftia*
1025	467	VII	870	Pseudomonas nitritireducens strain WZBFD3-5A2 16S ribosomal RNA, partial sequence	100%	0.0	99%	NR_133020.1	*Pseudomonas*
1029	467	II	300	Delftia tsuruhatensis strain NBRC 16741 16S ribosomal RNA gene, partial sequence	99%	4,00E-100	89%	NR_113870.1	*Comamonadaceae*
1040	467	V	655	Bacillus cereus ATCC 14579 16S ribosomal RNA (rrnA) gene, complete sequence	100%	0.0	99%	NR_074540.1	*Bacillus*
1042	462	V	681	Shigella boydii strain P288 16S ribosomal RNA gene, partial sequence	100%	0.0	99%	NR_104901.1	*Enterobacteriaceae*
1047	462	I	945	Enterococcus faecalis strain NBRC 100480 16S ribosomal RNA gene, partial sequence	100%	0.0	99%	NR_113901.1	*Enterococcus faecalis*
1048	462	I	1050	Enterobacter xiangfangensis strain	99%	0.0	99%	NR_126208.1	*Enterobacteriaceae*

				10-17 16S ribosomal RNA gene, partial sequence					
1071	466	VII	86	Delftia deserti strain YIM Y792 16S ribosomal RNA, partial sequence	91%	1,00E-35	100%	NR_136837.1	*Delftia*
1073	466	II	948	Bacillus anthracis strain ATCC 14578 16S ribosomal RNA gene, partial sequence	100%	0.0	99%	NR_041248.1	*Bacillus*
1110	388	II	356	Clostridium tertium strain JCM 6289 16S ribosomal RNA gene, partial sequence	100%	0.0	100%	NR_113325.1	*Clostridium tertium*
1120	508	VII	325	Clostridium tertium strain JCM 6289 16S ribosomal RNA gene, partial sequence	98%	5,00E-159	98%	NR_113325.1	*Clostridium*
1124	508	II	1085	Enterococcus faecium strain DSM 20477 16S ribosomal RNA gene, complete sequence	100%	0.0	99%	NR_114742.1	*Enterococcus*
1133	505	VII	1003	Enterococcus faecalis strain NBRC 100480 16S ribosomal RNA gene, partial sequence	100%	0.0	99%	NR_113901.1	*Enterococcus faecalis*
1153	388	V	400	Propionibacterium acnes strain ATCC 6919 16S ribosomal RNA, complete sequence	100%	0.0	99%	NR_040847.1	*Propionibacterium acnes*

1162	508	II	1005	Enterococcus faecium strain NBRC 100486 16S ribosomal RNA gene, partial sequence	98%	0.0	99%	NR_113904.1	*Enterococcus*
1164	508	VII	1026	Robinsoniella peoriensis strain PPC31 16S ribosomal RNA gene, complete sequence	100%	0.0	99%	NR_041882.1	*Robinsoniella peoriensis*
1185	516	IX	900	Enterococcus faecalis strain NBRC 100480 16S ribosomal RNA gene, partial sequence	100%	0.0	98%	NR_113901.1	*Enterococcus*
1188	471	IX	774	Pantoea eucrina strain LMG 2781 16S ribosomal RNA gene, partial sequence	97%	0.0	99%	NR_116246.1	*Pantoea eucrina*
1192	463	IX	337	Citrobacter koseri strain LMG 5519 16S ribosomal RNA gene, partial sequence	99%	5,00E-169	98%	NR_117751.1	*Enterobacteriaceae*
1202	504	VII	233	Clostridium tertium strain JCM 6289 16S ribosomal RNA gene, partial sequence	94%	2,00E-111	99%	NR_113325.1	*Clostridium tertium*
1295	501	II	826	Enterococcus faecium strain NBRC 100486 16S ribosomal RNA gene, partial sequence	99%	0.0	99%	NR_113904.1	*Enterococcus*
1319	501	II	224	Enterococcus faecium strain NBRC 100486 16S ribosomal RNA gene, partial sequence	98%	2,00E-112	100%	NR_113904.1	*Enterococcus*

1321	501	IX	1052	Clostridium septicum strain Pasteur III 16S ribosomal RNA gene, complete sequence	100%	0.0	98%	NR_026020.1	*Clostridium*
1340	519	IX	799	Pseudomonas nitroreducens strain NBRC 12694 16S ribosomal RNA gene, partial sequence	99%	0.0	99%	NR_113601.1	*Pseudomonas*
1343	519	X	323	Romboutsia sedimentorum strain LAM201 16S ribosomal RNA, partial sequence	90%	2,00E-132	96%	NR_134800.1	*Romboutsia*
1353	519	IV	220	Paenibacillus borealis strain KK19 16S ribosomal RNA gene, complete sequence	96%	2,00E-107	99%	NR_025299.1	*Paenibacillus borealis*
1359	502	IV	998	Enterococcus durans strain JCM 8725 16S ribosomal RNA gene, partial sequence	100%	0.0	99%	NR_113257.1	*Enterococcus*
1362	502	X	940	[Eubacterium] tenue strain ATCC 25553 16S ribosomal RNA gene, partial sequence	100%	0.0	99%	NR_115794.1	*Paeniclostridium tenue*
1368	460	X	843	Clostridium perfringens strain ATCC 13124 16S ribosomal RNA gene, complete sequence	99%	0.0	99%	NR_121697.1	*Clostridium perfringens*
1376	471	IV	1072	Bacillus kokeshiiformis strain MO-04 16S	99%	0.0	99%	NR_133975.1	*Bacillus kokeshiiformis*

				ribosomal RNA, partial sequence					
1377	516	VII	234	Clostridium tertium strain JCM 6289 16S ribosomal RNA gene, partial sequence	94%	1E-109	99%	NR_113325.1	*Clostridium tertium*
1381	509	VII	1037	Clostridium septicum strain Pasteur III 16S ribosomal RNA gene, complete sequence	100%	0.0	99%	NR_026020.1	*Clostridium septicum*
1388	463	VII	214	Clostridium perfringens strain JCM 1290 16S ribosomal RNA gene, partial sequence	98%	4,00E-103	98%	NR_113204.1	*Clostridium*
1393	675	VII	889	Clostridium perfringens strain JCM 1290 16S ribosomal RNA gene, partial sequence	100%	0.0	99%	NR_113204.1	*Clostridium perfringens*
1398	675	II	215	Enterococcus faecium strain NBRC 100486 16S ribosomal RNA gene, partial sequence	99%	1,00E-108	99%	NR_113904.1	*Enterococcus*
1404	463	VII	1074	Carnobacterium mobile strain DSM 4848 16S ribosomal RNA gene, partial sequence	99%	0.0	98%	NR_040926.1	*Carnobacterium*
1409	471	II	415	Enterococcus faecium strain NBRC 100486 16S ribosomal RNA gene, partial sequence	100%	0.0	99%	NR_113904.1	*Enterococcus*
1421	516	VII	774	[Clostridium] sordellii strain JCM 3814 16S	99%	0.0	98%	NR_113140.1	*Paeniclostridium*

		ribosomal RNA gene, partial sequence	

[1]Based on morphological (cell morphology, Gram, and endospore staining) and biochemical tests (catalase and oxidase tests).

[2] Phylotype assignment based on information from top three best matches displaying the higher nucleotide pairwise identity, using taxonomic threshold similarity values as discussed in Material and Methods chapter.

Supplementary Table 2 – Relative abundances, at the bacterial genus level, of each taxon in culturomics[1] and microbial profiling[2] approaches.

Taxon[3]	Culturomics	Microbial profiling
Bacillus	16	0
Blautia	0	7
Carnobacterium	3	4
Clostridioides	0	25
Clostridium	5	19
Collinsella	0	5
Delftia	3	0
Enterococcus	34	1
Escherichia	0	2
Fusobacterium	0	2
Lactobacillus	0	4
Lysinibacillus	4	0
Paeniclostridium	1	7
Paraclostridium	1	5
Pseudomonas	10	0
Ralstonia	5	0
Rummellibacillus	4	0
Sporosarcina	0	3
Others (<2%)	14	17

Taxon[3]	Female C	Male C	Female MP	Male MP
Bacillus	14	19	0	0
Blautia	0	0	8	6
Carnobacterium	4	2	6	0
Clostridioides	0	0	27	23
Clostridium	6	5	18	19
Collinsella	0	0	5	5
Delftia	1	4	0	0
Enterococcus	36	33	2	0
Escherichia	0	0	3	1
Fusobacterium	0	0	1	3
Lactobacillus	0	0	6	1
Lysinibacillus	5	3	0	0
Paeniclostridium	0	2	7	7
Paraclostridium	1	1	5	5
Pseudomonas	12	9	0	0
Ralstonia	3	7	0	0
Romboutsia	1	1	0	3
Rummellibacillus	2	6	0	0
Sporosarcina	0	1	0	7
Stenotrophomonas	1	2	0	0
Others (<2%)	15	7	12	16

Taxon[3]	Non-adults C	Adults C	Non-adults MP	Adults MP
Bacillus	17	16	0	0
Blautia	0	0	6	7
Carnobacterium	2	3	0	5
Clostridioides	0	0	37	22
Clostridium	11	4	18	19
Collinsella	0	0	3	5
Delftia	0	3	0	0
Enterococcus	29	36	0	1
Escherichia	0	0	3	2
Fusobacterium	0	0	0	3
Lactobacillus	0	0	0	4
Lysinibacillus	6	3	0	0
Paeniclostridium	2	1	4	8
Paraclostridium	0	2	3	5
Pseudomonas	9	11	0	0
Ralstonia	5	5	0	0
Romboutsia	2	1	0	2
Rummellibacillus	9	3	0	0
Sporosarcina	2	0	0	4
Others (<2%)	9	13	16	13

[1]the relative abundances (%) were calculated based on the differentiation information obtained by Random Amplification of Polymorphic DNA (RAPD) and 16S rRNA gene sequencing; the isolates within each RAPD cluster were considered to belong to the same genus of the isolate(s) within that cluster that was(were) subject to molecular identification; calculations were made by dividing the number of differentiated strains within each cluster by the total number of differentiated strains using RAPD.

[2]the relative abundances (%) were calculated based on the taxonomical classification of OTU and the total number of obtained OTU reads.

[3]taxon representativity was based on the relative abundance of groups (%).

Supplementary Table 3 – Information on the ITS nucleotide sequences of a selected group of fungi isolates.

**Isolate**	**Animal**	**Phenotypic Identification**[1]	**Nucleotide Sequence Length**	**Closest Reference Sequence Match**	**Query cover**	**E-value**	**Nucleotide Sequence Identity**	**Accession Number**	**Assigned Phylotype**[2]
1205	471	XIII	400	Pseudozyma sp. JS1231 18S rRNA gene (partial), ITS1, 5.8S rRNA gene, ITS2 and 26S rRNA gene (partial), isolate 1231	80%	5,00E-134	94%	AM176740.1	*Pseudozyma*
1206	675	XIII	400	Mucor circinelloides strain S032IMR 18S ribosomal RNA gene, partial sequence; internal transcribed spacer 1 and 5.8S ribosomal RNA gene, complete sequence; and internal transcribed spacer 2, partial sequence	100%	0.0	99%	KU198340.1	*Mucor circinelloides*
1212	509	XII	459	Cryptococcus albidus var. kuetzingii culture-collection CBS:6086 large subunit ribosomal RNA gene, partial sequence	98%	0.0	99%	KY106964.1	*Cryptococcus albidus/ Naganishia albida*
1213	463	XII	400	Cryptococcus albidus var. kuetzingii strain YM26709 26S ribosomal RNA gene, partial sequence	62%	1,00E-105	95%	KY463404.1	*Cryptococcus albidus/ Naganishia albida*
1331	460	XIII	400	Penicillium citreonigrum strain SFCF20120912-25 18S ribosomal RNA gene, partial sequence; internal transcribed spacer 1, 5.8S ribosomal RNA gene, and internal transcribed spacer 2, complete sequence; and 28S ribosomal RNA gene, partial sequence	90%	2,00E-163	96%	KF313080.1	*Penicillium*

1334	463	XIII	482	Penicillium amaliae strain CV401 18S ribosomal RNA gene, partial sequence; internal transcribed spacer 1, 5.8S ribosomal RNA gene, and internal transcribed spacer 2, complete sequence; and 28S ribosomal RNA gene, partial sequence	100%	0.0	99%	JX091440.1	*Penicillium amaliae*

[1]Based on morphological (hyphal septation and spores color, morphology, and septation, cell morphology, and division) tests.
[2] Phylotype assignment based on information from top three best matches displaying the higher nucleotide pairwise identity, using taxonomic threshold similarity values as discussed in Material and Methods chapter.

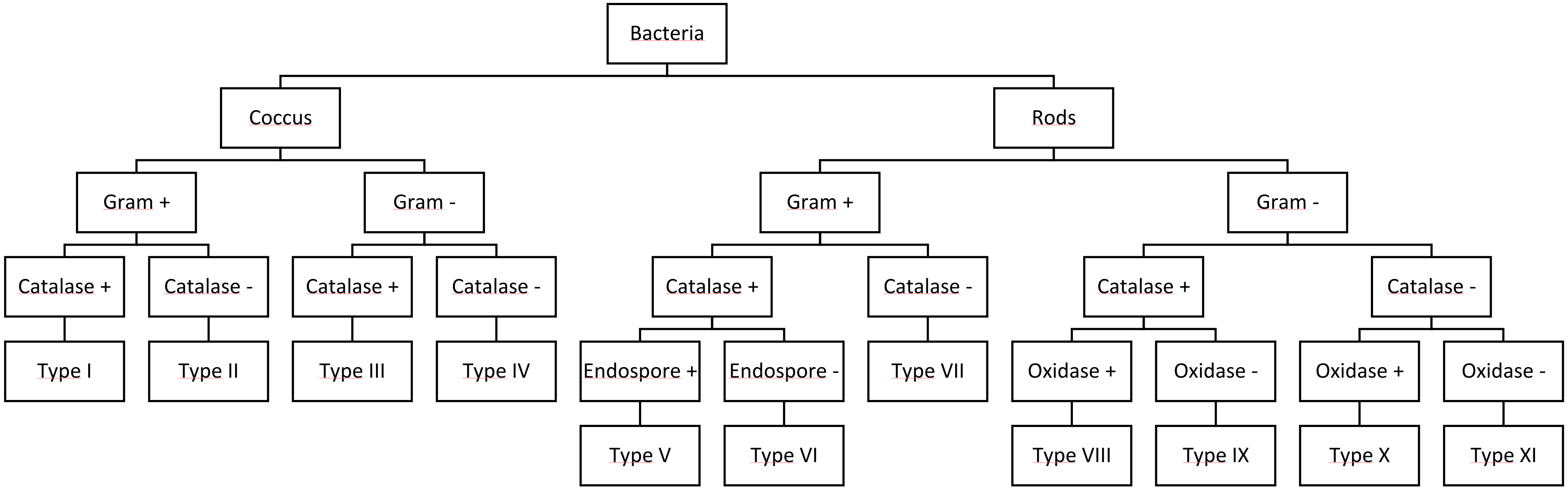

Supplementary Figure 1 – Flowchart used for differentiation of the purified bacterial isolates into different morpho-physiological types (from [25]).

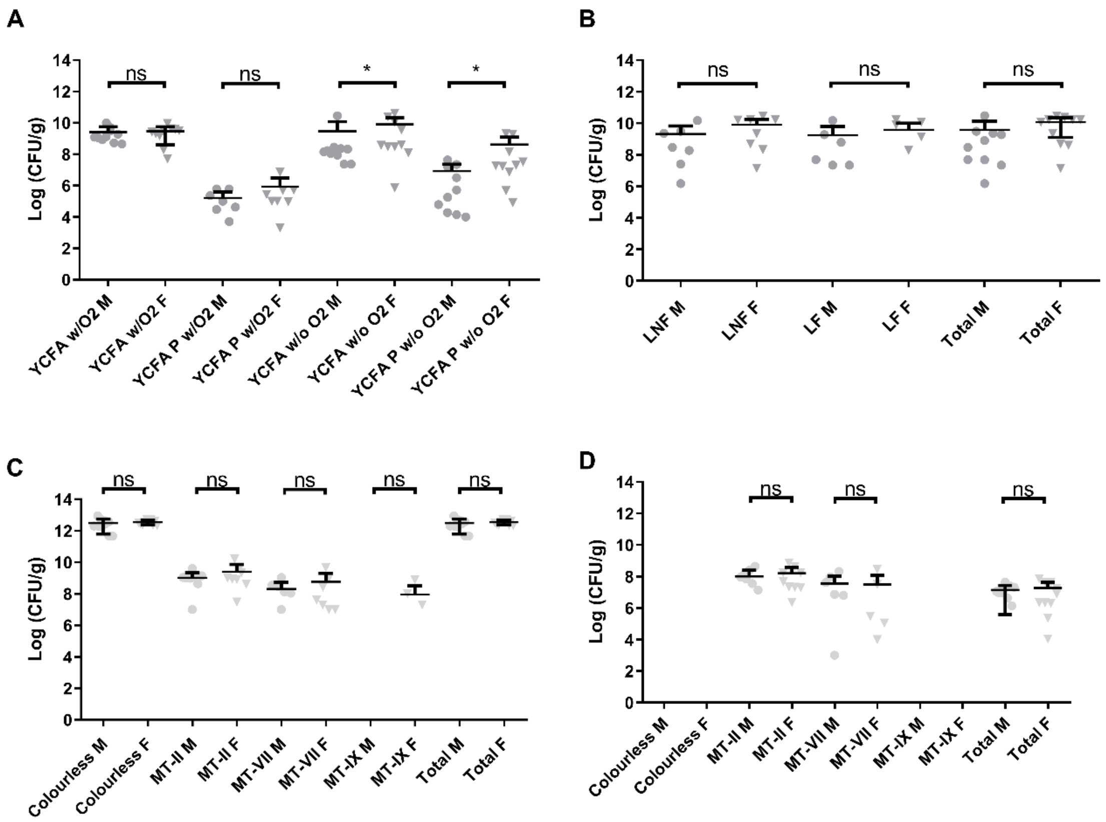

Supplementary Figure 2 — Microbial load (expressed as Log CFU/mL) of mongoose cultivable bacteria grown in selective and non-selective media. (A) Comparison among four different media/conditions: YCFA incubated under aerobiosis (YCFA w/O2), YCFA supplemented with sodium taurocholate and incubated under aerobiosis (YCFA P w/O2), YCFA incubated under anaerobiosis (YCFA w/o O2), and YCFA supplemented with sodium taurocholate and incubated under anaerobiosis (YCFA P w/o O2). (B) Comparison among lactose non-fermenting (LNF) bacteria and lactose-fermenting (LF) bacteria in MacConkey medium. (C/D) Comparison between Extended-spectrum beta-lactamases (ESBL) Chromogenic medium (C) without (ESBL w/o AS) and (D) with (ESBL w/ AS) ESBL antibiotic supplement with the results presented by colony color/type. Results from male (M), female (F), and in total (T) are presented. Horizontal bars represent the mean and error bars represent the standard deviation from 10 (male and female) and 20 (total) independent values. Statistical analysis was performed using a Mann-Whitney test (α=0.05). ns – non-significant (p-value≥ 0.05); * - significant (p-value<0.05).

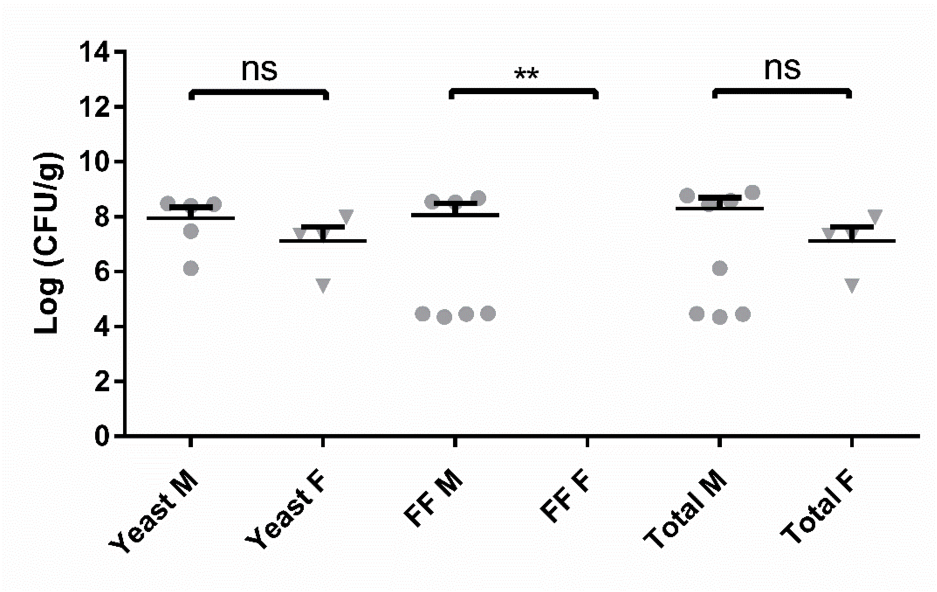

Supplementary Figure 3 – Comparison of microbial load (expressed as Log CFU/g) between male and female individuals in Potato Dextrose Agar medium with chloramphenicol (PDA w/ CHLO). Results from yeast and filamentous fungi (FF) are presented. Horizontal bars represent the mean and error bars represent the standard deviation from 10 independent values. Statistical analysis was performed using a Mann-Whitney test (α=0.05); ns – non-significant (p-value≥ 0.05), ** - very significant (p-value=0.001 to 0.01).

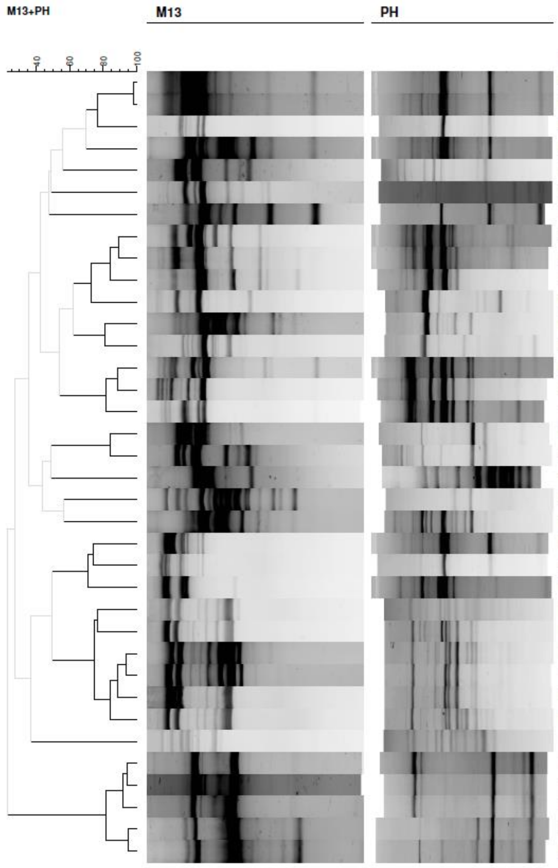

Isolate	Host	Medium	Oxygen	MT	Sex	Age	Phylotype
0003	HI462	MacConkey	Y	IX	Female	Adult	
0004	HI462	MacConkey	Y	IX	Female	Adult	
0058	HI462	ESBL w/o AS	Y	IX	Female	Adult	
0024	HI462	YCFA	N	IX	Female	Adult	
0271	HI396	ESBL w/o AS	Y	IX	Female	Adult	
0670	HI467	ESBL w/o AS	Y	IX	Male	Adult	
1042	HI462	YCFA	Y	V	Female	Adult	Enterobacteriaceae
0039	HI399	YCFA	N	IX	Female	Adult	
0205	HI399	MacConkey	Y	IX	Female	Adult	Hafniaceae
0273	HI399	ESBL w/o AS	Y	IX	Female	Adult	
0839	HI388	YCFA	Y	IX	Female	Sub-adult	
0824	HI502	MacConkey	Y	IX	Male	Juvenile 2	
0848	HI460	YCFA	Y	IX	Male	Juvenile 2	
0005	HI462	MacConkey	Y	IX	Female	Adult	
0300	HI462	MacConkey	Y	IX	Female	Adult	
1048	HI462	YCFA	Y	I	Female	Adult	Enterobacteriaceae
0490	HI504	ESBL w/o AS	Y	IX	Female	Sub-adult	
0496	HI505	ESBL w/o AS	Y	IX	Female	Adult	
1192	HI463	MacConkey	Y	IX	Male	Adult	
0816	HI502	MacConkey	Y	IX	Male	Juvenile 2	
1188	HI471	MacConkey	Y	IX	Male	Adult	Pantoea eucrina
0195	HI462	MacConkey	Y	IX	Female	Adult	
0248	HI462	ESBL w/o AS	Y	IX	Female	Adult	Enterobacteriaceae
0029	HI462	YCFA	N	V	Female	Adult	Enterobacteriaceae
1174	HI675	MacConkey	Y	IX	Male	Adult	
1183	HI516	MacConkey	Y	IX	Male	Adult	
1181	HI471	MacConkey	Y	IX	Male	Adult	
1200	HI636	MacConkey	Y	IX	Female (L)	Adult	
1193	HI501	MacConkey	Y	IX	Female	Adult	
1175	HI509	MacConkey	Y	IX	Male	Adult	
1224	HI675	YCFA	N	II	Male	Adult	
1020	HI383	YCFA	Y	VIII	Male	Adult	Enterobacteriaceae
1070	HI466	YCFA	Y	VII	Male	Adult	
1055	HI396	YCFA	Y	VII	Female	Adult	
0228	HI467	ESBL w/o AS	Y	VIII	Male	Adult	
0520	HI505	ESBL w/o AS	Y	XI	Female	Adult	

Supplementary Figure 4 – Bacterial isolates identification and differentiation by a hierarchical numerical analysis. The PCR fingerprints obtained for M13 and PH were integrated, similarity was calculated by Pearson correlation coefficient and clustering was performed with UPGMA. The scale corresponds to global percentage of similarity. Cut-off value for cluster formation at 70% similarity. N – No; Y – Yes; (L) – Lactating.

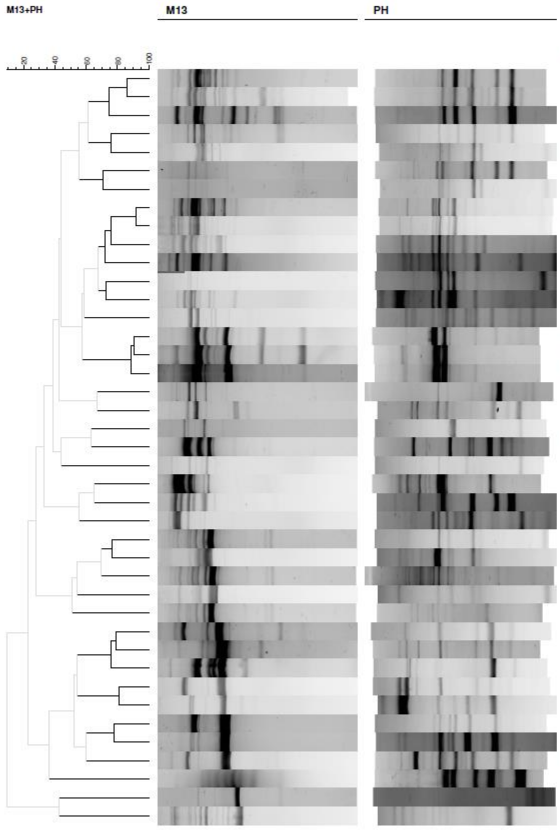

Isolate	Host	Medium	Oxygen	MT	Sex	Age	Phylotype
1112	HI388	YCFA P	N	VII	Female	Sub-adult	
1120	HI508	YCFA P	N	VII	Female	Adult	Clostridium sp.
1310	HI636	YCFA	N	VII	Female (L)	Adult	
0117	HI636	YCFA P	N	VII	Female (L)	Adult	Clostridium tertium
1144	HI388	YCFA	N	VII	Female	Sub-adult	
1202	HI504	YCFA P	N	VII	Female	Sub-adult	Clostridium tertium
1138	HI504	YCFA	N	VII	Female	Sub-adult	
1286	HI636	YCFA P	N	VII	Female (L)	Adult	
1299	HI501	YCFA P	N	VII	Female	Adult	
1393	HI675	YCFA P	N	VII	Male	Adult	Clostridium perfringers
1368	HI460	YCFA	N	X	Male	Juvenile 2	Clostridium perfringens
1321	HI501	YCFA	N	VII	Female	Adult	Clostridium sp.
1367	HI460	YCFA	N	VII	Male	Juvenile 2	
1388	HI463	YCFA P	N	VII	Male	Adult	Clostridium sp.
0392	HI509	ESBL w/o AS	Y	VII	Male	Adult	
0593	HI388	ESBL w/o AS	Y	VII	Female	Sub-adult	
0583	HI471	ESBL w/o AS	Y	VII	Male	Adult	
0054	HI383	YCFA	N	IX	Male	Adult	Paeniclostridium sp.
0084	HI462	YCFA P	N	VII	Female	Adult	Peptoclostridium bifermentans
0101	HI519	YCFA P	N	VII	Female	Adult	Peptoclostridium bifermentans
1025	HI467	YCFA	Y	VII	Male	Adult	
0131	HI516	YCFA P	N	VII	Male	Adult	Peptoclostridium bifermentans
0349	HI462	YCFA P	N	VII	Female	Adult	
1377	HI516	YCFA P	N	VII	Male	Adult	Clostridium tertium
1381	HI509	YCFA P	N	VII	Male	Adult	Clostridium septicum
0072	HI467	YCFA P	N	VII	Male	Adult	Peptostreptococcaceae
1428	HI675	YCFA	N	VII	Male	Adult	
0043	HI467	YCFA	N	IX	Male	Adult	Clostridiales
1421	HI516	YCFA	N	VII	Male	Adult	Paeniclostridium sp.
0009	HI467	ESBL w/ AS	Y	VII	Male	Adult	Peptostreptococcaceae
0263	HI396	ESBL w/o AS	Y	VII	Female	Adult	
0368	HI504	ESBL w/o AS	Y	VII	Female	Sub-adult	
0506	HI508	ESBL w/o AS	Y	XI	Female	Adult	Carnobacterium sp.
0653	HI462	ESBL w/o AS	Y	VII	Female	Adult	
1400	HI509	YCFA	N	VII	Male	Adult	
0104	HI519	YCFA P	N	X	Female	Adult	Clostridium perfringens
1335	HI519	YCFA P	N	VII	Female	Adult	
1110	HI388	YCFA P	N	II	Female	Sub-adult	Clostridium tertium
1362	HI502	YCFA	N	X	Male	Juvenile 2	Paeniclostridium tenue
0659	HI383	ESBL w/o AS	Y	IX	Male	Adult	
1404	HI463	YCFA	N	VII	Male	Adult	Carnobacterium sp.

Supplementary Figure 4 (Continuation)

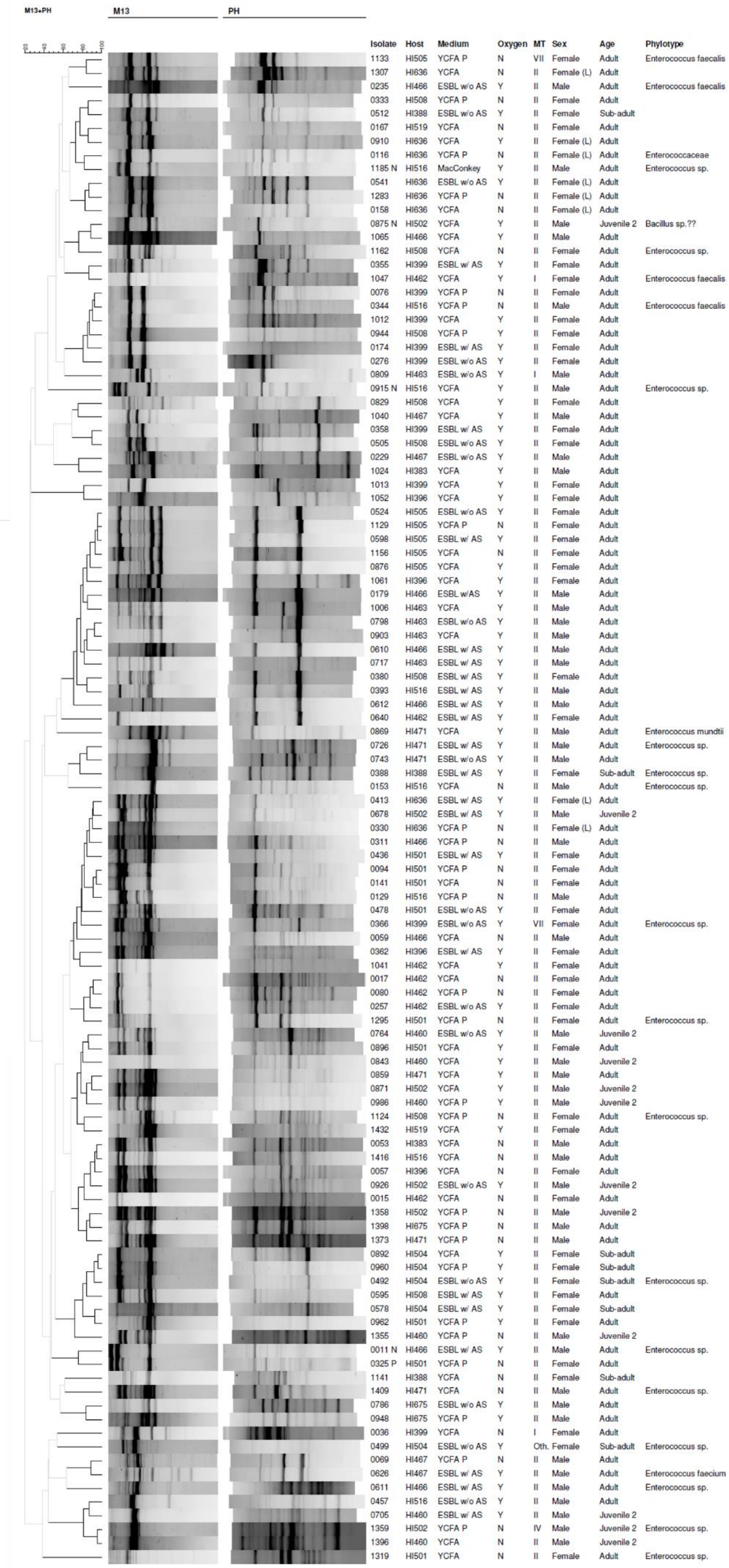

Isolate	Host	Medium	Oxygen	MT	Sex	Age	Phylotype
1133	HI505	YCFA P	N	VII	Female	Adult	Enterococcus faecalis
1307	HI636	YCFA	N	II	Female (L)	Adult	
0235	HI466	ESBL w/o AS	Y	II	Male	Adult	Enterococcus faecalis
0333	HI508	YCFA P	N	II	Female	Adult	
0512	HI388	ESBL w/o AS	Y	II	Female	Sub-adult	
0167	HI519	YCFA	N	II	Female	Adult	
0910	HI636	YCFA	Y	II	Female (L)	Adult	
0116	HI636	YCFA P	N	II	Female (L)	Adult	Enterococcaceae
1185 N	HI516	MacConkey	Y	II	Male	Adult	Enterococcus sp.
0541	HI636	ESBL w/o AS	Y	II	Female (L)	Adult	
1283	HI636	YCFA P	N	II	Female (L)	Adult	
0158	HI636	YCFA	N	II	Female (L)	Adult	
0875 N	HI502	YCFA	Y	II	Male	Juvenile 2	Bacillus sp.??
1065	HI466	YCFA	Y	II	Male	Adult	
1162	HI508	YCFA	N	II	Female	Adult	Enterococcus sp.
0355	HI399	ESBL w/ AS	Y	II	Female	Adult	
1047	HI462	YCFA	Y	I	Female	Adult	Enterococcus faecalis
0076	HI399	YCFA P	N	II	Female	Adult	
0344	HI516	YCFA P	N	II	Male	Adult	Enterococcus faecalis
1012	HI399	YCFA	Y	II	Female	Adult	
0944	HI508	YCFA P	Y	II	Female	Adult	
0174	HI399	ESBL w/ AS	Y	II	Female	Adult	
0276	HI399	ESBL w/o AS	Y	II	Female	Adult	
0809	HI463	ESBL w/o AS	Y	I	Male	Adult	
0915 N	HI516	YCFA	Y	II	Male	Adult	Enterococcus sp.
0829	HI508	YCFA	Y	II	Female	Adult	
1040	HI467	YCFA	Y	II	Male	Adult	
0358	HI399	ESBL w/ AS	Y	II	Female	Adult	
0505	HI508	ESBL w/o AS	Y	II	Female	Adult	
0229	HI467	ESBL w/o AS	Y	II	Male	Adult	
1024	HI383	YCFA	Y	II	Male	Adult	
1013	HI399	YCFA	Y	II	Female	Adult	
1052	HI396	YCFA	Y	II	Female	Adult	
0524	HI505	ESBL w/o AS	Y	II	Female	Adult	
1129	HI505	YCFA P	N	II	Female	Adult	
0598	HI505	ESBL w/ AS	Y	II	Female	Adult	
1156	HI505	YCFA	N	II	Female	Adult	
0876	HI505	YCFA	Y	II	Female	Adult	
1061	HI396	YCFA	Y	II	Female	Adult	
0179	HI466	ESBL w/AS	Y	II	Male	Adult	
1006	HI463	YCFA	Y	II	Male	Adult	
0798	HI463	ESBL w/o AS	Y	II	Male	Adult	
0903	HI463	YCFA	Y	II	Male	Adult	
0610	HI466	ESBL w/ AS	Y	II	Male	Adult	
0717	HI463	ESBL w/ AS	Y	II	Male	Adult	
0380	HI508	ESBL w/ AS	Y	II	Female	Adult	
0393	HI516	ESBL w/ AS	Y	II	Male	Adult	
0612	HI466	ESBL w/ AS	Y	II	Male	Adult	
0640	HI462	ESBL w/ AS	Y	II	Female	Adult	
0869	HI471	YCFA	Y	II	Male	Adult	Enterococcus mundtii
0726	HI471	ESBL w/ AS	Y	II	Male	Adult	Enterococcus sp.
0743	HI471	ESBL w/o AS	Y	II	Male	Adult	
0388	HI388	ESBL w/ AS	Y	II	Female	Sub-adult	Enterococcus sp.
0153	HI516	YCFA	N	II	Male	Adult	Enterococcus sp.
0413	HI636	ESBL w/ AS	Y	II	Female (L)	Adult	
0678	HI502	ESBL w/ AS	Y	II	Male	Juvenile 2	
0330	HI636	YCFA P	N	II	Female (L)	Adult	
0311	HI466	YCFA P	N	II	Male	Adult	
0436	HI501	ESBL w/ AS	Y	II	Female	Adult	
0094	HI501	YCFA P	N	II	Female	Adult	
0141	HI501	YCFA	N	II	Female	Adult	
0129	HI516	YCFA P	N	II	Male	Adult	
0478	HI501	ESBL w/o AS	Y	II	Female	Adult	
0366	HI399	ESBL w/o AS	Y	VII	Female	Adult	Enterococcus sp.
0059	HI466	YCFA	N	II	Male	Adult	
0362	HI396	ESBL w/ AS	Y	II	Female	Adult	
1041	HI462	YCFA	Y	II	Female	Adult	
0017	HI462	YCFA	N	II	Female	Adult	
0080	HI462	YCFA P	N	II	Female	Adult	
0257	HI462	ESBL w/o AS	Y	II	Female	Adult	
1295	HI501	YCFA P	N	II	Female	Adult	Enterococcus sp.
0764	HI460	ESBL w/o AS	Y	II	Male	Juvenile 2	
0896	HI501	YCFA	Y	II	Female	Adult	
0843	HI460	YCFA	Y	II	Male	Juvenile 2	
0859	HI471	YCFA	Y	II	Male	Adult	
0871	HI502	YCFA	Y	II	Male	Juvenile 2	
0986	HI460	YCFA P	Y	II	Male	Juvenile 2	
1124	HI508	YCFA P	N	II	Female	Adult	Enterococcus sp.
1432	HI519	YCFA	Y	II	Female	Adult	
0053	HI383	YCFA	N	II	Male	Adult	
1416	HI516	YCFA	N	II	Male	Adult	
0057	HI396	YCFA	N	II	Female	Adult	
0926	HI502	ESBL w/o AS	Y	II	Male	Juvenile 2	
0015	HI462	YCFA	N	II	Female	Adult	
1358	HI502	YCFA P	N	II	Male	Juvenile 2	
1398	HI675	YCFA P	N	II	Male	Adult	
1373	HI471	YCFA P	N	II	Male	Adult	
0892	HI504	YCFA	Y	II	Female	Sub-adult	
0960	HI504	YCFA P	Y	II	Female	Sub-adult	
0492	HI504	ESBL w/o AS	Y	II	Female	Sub-adult	Enterococcus sp.
0595	HI508	ESBL w/ AS	Y	II	Female	Adult	
0578	HI504	ESBL w/ AS	Y	II	Female	Sub-adult	
0962	HI501	YCFA P	Y	II	Female	Adult	
1355	HI460	YCFA P	N	II	Male	Juvenile 2	
0011 N	HI466	ESBL w/ AS	Y	II	Male	Adult	Enterococcus sp.
0325 P	HI501	YCFA P	N	II	Female	Adult	
1141	HI388	YCFA	N	II	Female	Sub-adult	
1409	HI471	YCFA	N	II	Male	Adult	Enterococcus sp.
0786	HI675	ESBL w/o AS	Y	II	Male	Adult	
0948	HI675	YCFA P	Y	II	Male	Adult	
0036	HI399	YCFA	N	I	Female	Adult	
0499	HI504	ESBL w/o AS	Y	Oth.	Female	Sub-adult	Enterococcus sp.
0069	HI467	YCFA P	N	II	Male	Adult	
0626	HI467	ESBL w/ AS	Y	II	Male	Adult	Enterococcus faecium
0611	HI466	ESBL w/ AS	Y	II	Male	Adult	Enterococcus sp.
0457	HI516	ESBL w/o AS	Y	II	Male	Adult	
0705	HI460	ESBL w/ AS	Y	II	Male	Juvenile 2	
1359	HI502	YCFA P	N	IV	Male	Juvenile 2	Enterococcus sp.
1396	HI460	YCFA	N	II	Male	Juvenile 2	
1319	HI501	YCFA	N	II	Female	Adult	Enterococcus sp.

9

Supplementary Figure 4 (Continuation)

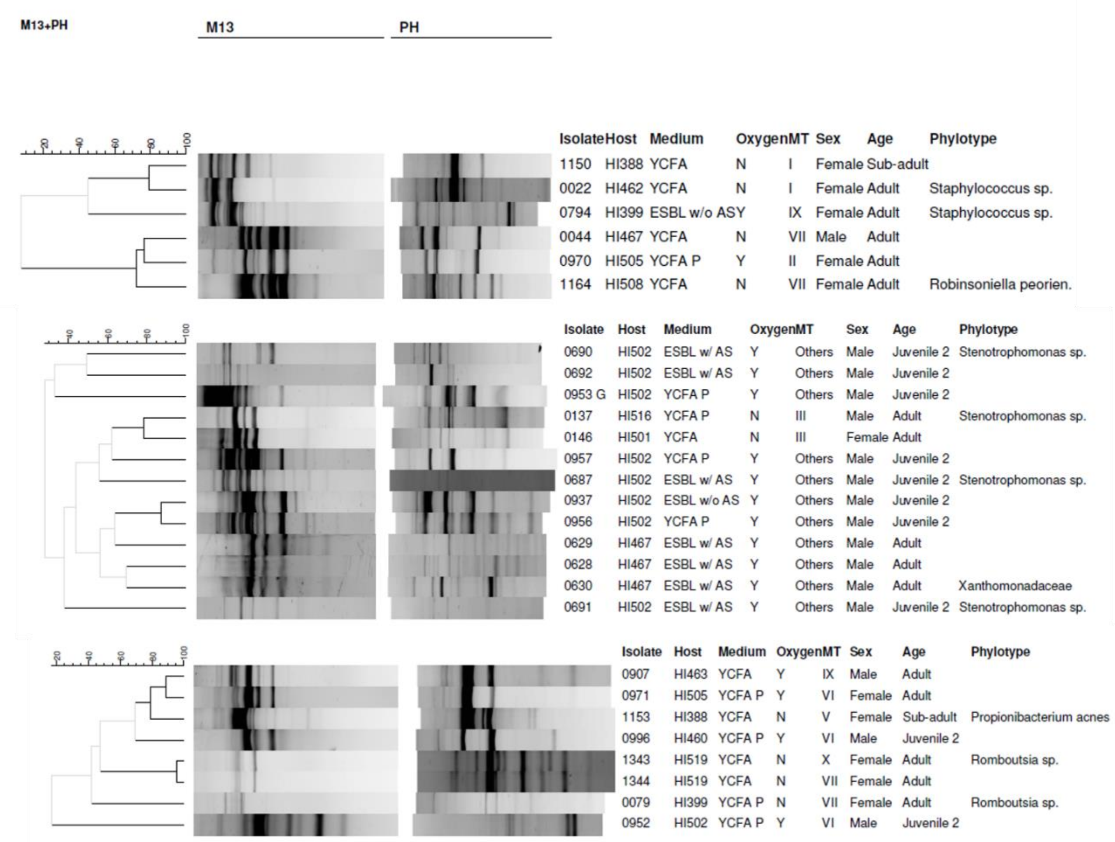

Supplementary Figure 4 (Continuation)

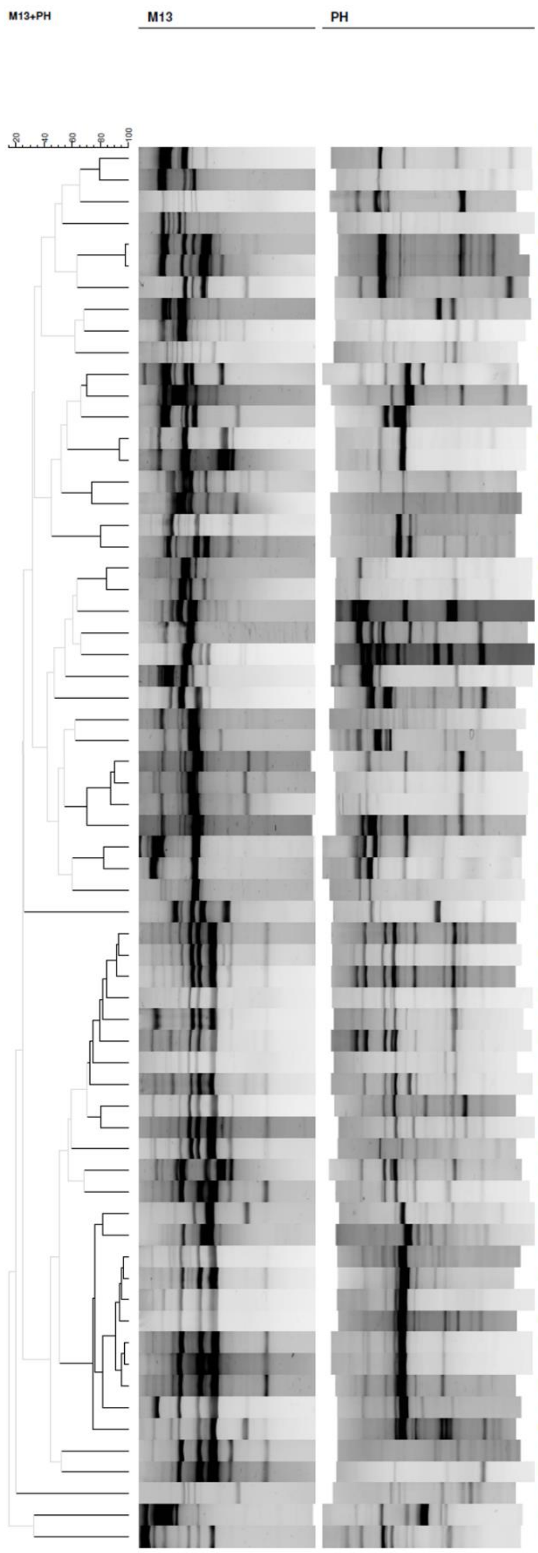

Isolate	Host	Medium	Oxygen	MT	Sex	Age	Phylotype
0462	HI516	ESBL w/o AS	Y	VIII	Male	Adult	Rummeliibacillus stabekisii
0964	HI504	YCFA P	Y	VI	Female	Sub-adult	
0340	HI516	YCFA P	N	VII	Male	Adult	Bacillus sp.
0864	HI471	YCFA	Y	VIII	Male	Adult	Lysinibacillus sp.
0746	HI471	ESBL S/ab	Y	V	Male	Adult	Rummeliibacillus stabekisii
1064	HI466	YCFA	Y	V	Male	Adult	
0919	HI463	ESBL w/o AS	Y	VI	Male	Adult	Rummeliibacillus stabekisii
0889	HI504	YCFA	Y	VIII	Female	Sub-adult	Lysinibacillus sp.
1001	HI388	YCFA P	Y	VIII	Female	Sub-adult	Lysinibacillus sp.
0147	HI501	YCFA	N	VII	Female	Adult	Bacillus sp.
0596 N	HI508	ESBL w/o AS	Y	VIII	Female	Adult	Bacillus sp.
1004	HI388	YCFA P	Y	VI	Female	Sub-adult	Psychrobacillus soli
0879	HI505	YCFA	Y	VIII	Female	Adult	Lysinibacillus sp.
0998	HI516	YCFA P	Y	IX	Male	Adult	Bacillaceae
1000	HI388	YCFA P	Y	IX	Female	Sub-adult	
0680	HI502	ESBL w/ AS	Y	Others	Male	Juvenile 2	
0955	HI502	YCFA P	Y	V	Male	Juvenile 2	
0383	HI509	ESBL w/o AS	Y	X	Male	Adult	
0390	HI509	ESBL w/o AS	Y	XI	Male	Adult	Bacillus anthracis
0963	HI504	YCFA P	Y	VI	Female	Sub-adult	Rummeliibacillus stabekisii
0965	HI501	YCFA P	Y	VI	Female	Adult	
1376	HI471	YCFA P	N	IV	Male	Adult	Bacillus kokeshiiformis
0923	HI463	ESBL w/o AS	Y	VIII	Male	Adult	Lysinibacillus sp.
1353	HI519	YCFA	N	IV	Female	Adult	Paenibacillus borealis
0456	HI516	ESBL w/o AS	Y	Others	Male	Adult	Rummeliibacillus stabekisii
0559	HI388	ESBL w/o AS	Y	X	Female	Sub-adult	Lysinibacillus fusiformis
0302	HI396	YCFA P	N	IX	Female	Adult	Bacillus sp.
0338	HI519	YCFA P	N	VII	Female	Adult	Bacillus sp.
0943	HI466	YCFA P	Y	VI	Male	Adult	
0982	HI396	YCFA P	Y	VI	Female	Adult	
0984	HI636	YCFA P	Y	VI	Female (L)	Adult	
1073	HI466	YCFA	Y	II	Male	Adult	Bacillus sp.
0928 N	HI502	ESBL w/o AS	Y	VI	Male	Juvenile 2	Bacillaceae
0942 N	HI466	YCFA P	Y	VII	Male	Adult	Bacillus sp.
0308	HI383	YCFA P	N	IX	Male	Adult	
0509	HI508	ESBL w/o AS	Y	IX	Female	Adult	Lysinibacillus sp.
0359	HI519	ESBL w/ AS	Y	VII	Female	Adult	
0507	HI508	ESBL w/o AS	Y	VII	Female	Adult	
0377	HI508	ESBL w/ AS	Y	VII	Female	Adult	
0410	HI516	ESBL w/ AS	Y	VII	Male	Adult	
0091	HI501	YCFA P	N	IX	Female	Adult	Bacillus anthracis
0464	HI516	ESBL w/o AS	Y	VII	Male	Adult	
0149	HI516	YCFA	N	VII	Male	Adult	
0428	HI501	ESBL w/ AS	Y	VII	Female	Adult	
0562	HI388	ESBL w/ AS	Y	VII	Female	Sub-adult	
0837	HI508	YCFA	Y	V	Female	Adult	Bacillus sp.
0320	HI501	YCFA P	N	VII	Female	Adult	Bacillus sp.
0589	HI504	ESBL w/ AS	Y	VII	Female	Sub-adult	Bacillus sp.
0978	HI471	YCFA P	Y	VI	Male	Adult	Bacillus sp.
0828	HI509	YCFA	Y	V	Male	Adult	
1430	HI519	YCFA P	Y	V	Female	Adult	
0714	HI463	ESBL w/ AS	Y	VII	Male	Adult	
0805	HI463	ESBL w/o AS	Y	VII	Male	Adult	Bacillus anthracis
0855	HI675	YCFA	Y	VI	Male	Adult	
0776	HI675	ESBL w/o AS	Y	VII	Male	Adult	
0415	HI675	ESBL w/ AS	Y	VII	Male	Adult	
0417	HI505	ESBL w/o AS	Y	VII	Female	Adult	
0544	HI636	ESBL w/o AS	Y	VII	Female (L)	Adult	
0699	HI509	ESBL w/ AS	Y	VII	Male	Adult	
0788	HI509	ESBL w/o AS	Y	VII	Male	Adult	
0734	HI471	ESBL w/ AS	Y	VII	Male	Adult	Bacillus sp.
0868	HI471	YCFA	Y	VI	Male	Adult	
0531	HI505	ESBL w/o AS	Y	V	Female	Adult	Lysinibacillus sp.
0246 N	HI466	ESBL w/o AS	Y	V	Male	Adult	Lysinibacillus sp.
0325 G	HI501	YCFA P	N	V	Female	Adult	Bacillaceae

Supplementary Figure 4 (Continuation)

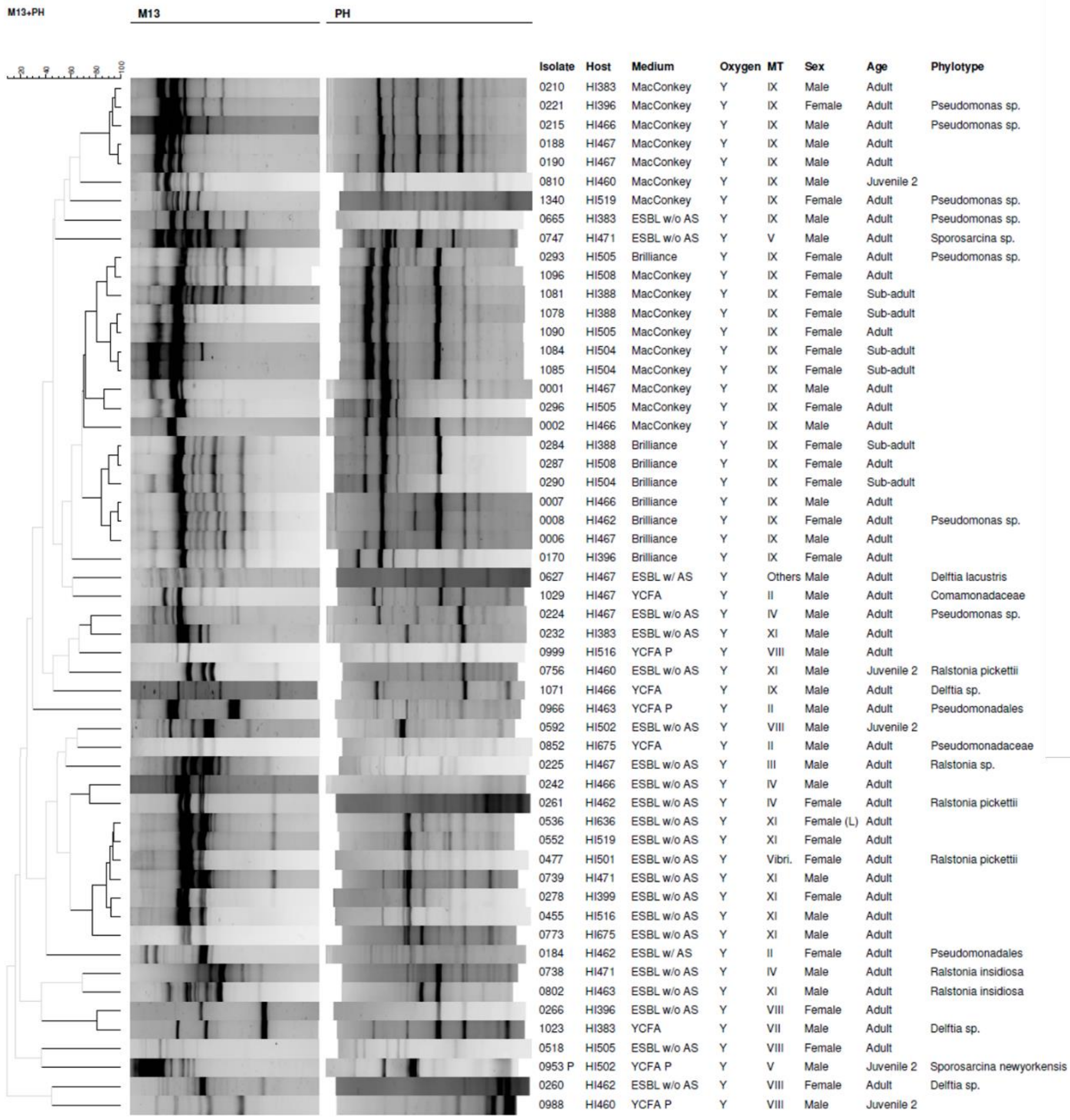

Isolate	Host	Medium	Oxygen	MT	Sex	Age	Phylotype
0210	HI383	MacConkey	Y	IX	Male	Adult	
0221	HI396	MacConkey	Y	IX	Female	Adult	Pseudomonas sp.
0215	HI466	MacConkey	Y	IX	Male	Adult	Pseudomonas sp.
0188	HI467	MacConkey	Y	IX	Male	Adult	
0190	HI467	MacConkey	Y	IX	Male	Adult	
0810	HI460	MacConkey	Y	IX	Male	Juvenile 2	
1340	HI519	MacConkey	Y	IX	Female	Adult	Pseudomonas sp.
0665	HI383	ESBL w/o AS	Y	IX	Male	Adult	Pseudomonas sp.
0747	HI471	ESBL w/o AS	Y	V	Male	Adult	Sporosarcina sp.
0293	HI505	Brilliance	Y	IX	Female	Adult	Pseudomonas sp.
1096	HI508	MacConkey	Y	IX	Female	Adult	
1081	HI388	MacConkey	Y	IX	Female	Sub-adult	
1078	HI388	MacConkey	Y	IX	Female	Sub-adult	
1090	HI505	MacConkey	Y	IX	Female	Adult	
1084	HI504	MacConkey	Y	IX	Female	Sub-adult	
1085	HI504	MacConkey	Y	IX	Female	Sub-adult	
0001	HI467	MacConkey	Y	IX	Male	Adult	
0296	HI505	MacConkey	Y	IX	Female	Adult	
0002	HI466	MacConkey	Y	IX	Male	Adult	
0284	HI388	Brilliance	Y	IX	Female	Sub-adult	
0287	HI508	Brilliance	Y	IX	Female	Adult	
0290	HI504	Brilliance	Y	IX	Female	Sub-adult	
0007	HI466	Brilliance	Y	IX	Male	Adult	
0008	HI462	Brilliance	Y	IX	Female	Adult	Pseudomonas sp.
0006	HI467	Brilliance	Y	IX	Male	Adult	
0170	HI396	Brilliance	Y	IX	Female	Adult	
0627	HI467	ESBL w/ AS	Y	Others	Male	Adult	Delftia lacustris
1029	HI467	YCFA	Y	II	Male	Adult	Comamonadaceae
0224	HI467	ESBL w/o AS	Y	IV	Male	Adult	Pseudomonas sp.
0232	HI383	ESBL w/o AS	Y	XI	Male	Adult	
0999	HI516	YCFA P	Y	VIII	Male	Adult	
0756	HI460	ESBL w/o AS	Y	XI	Male	Juvenile 2	Ralstonia pickettii
1071	HI466	YCFA	Y	IX	Male	Adult	Delftia sp.
0966	HI463	YCFA P	Y	II	Male	Adult	Pseudomonadales
0592	HI502	ESBL w/o AS	Y	VIII	Male	Juvenile 2	
0852	HI675	YCFA	Y	II	Male	Adult	Pseudomonadaceae
0225	HI467	ESBL w/o AS	Y	III	Male	Adult	Ralstonia sp.
0242	HI466	ESBL w/o AS	Y	IV	Male	Adult	
0261	HI462	ESBL w/o AS	Y	IV	Female	Adult	Ralstonia pickettii
0536	HI636	ESBL w/o AS	Y	XI	Female (L)	Adult	
0552	HI519	ESBL w/o AS	Y	XI	Female	Adult	
0477	HI501	ESBL w/o AS	Y	Vibri.	Female	Adult	Ralstonia pickettii
0739	HI471	ESBL w/o AS	Y	XI	Male	Adult	
0278	HI399	ESBL w/o AS	Y	XI	Female	Adult	
0455	HI516	ESBL w/o AS	Y	XI	Male	Adult	
0773	HI675	ESBL w/o AS	Y	XI	Male	Adult	
0184	HI462	ESBL w/ AS	Y	II	Female	Adult	Pseudomonadales
0738	HI471	ESBL w/o AS	Y	IV	Male	Adult	Ralstonia insidiosa
0802	HI463	ESBL w/o AS	Y	XI	Male	Adult	Ralstonia insidiosa
0266	HI396	ESBL w/o AS	Y	VIII	Female	Adult	
1023	HI383	YCFA	Y	VII	Male	Adult	Delftia sp.
0518	HI505	ESBL w/o AS	Y	VIII	Female	Adult	
0953 P	HI502	YCFA P	Y	V	Male	Juvenile 2	Sporosarcina newyorkensis
0260	HI462	ESBL w/o AS	Y	VIII	Female	Adult	Delftia sp.
0988	HI460	YCFA P	Y	VIII	Male	Juvenile 2	

Supplementary Figure 4 (Continuation)

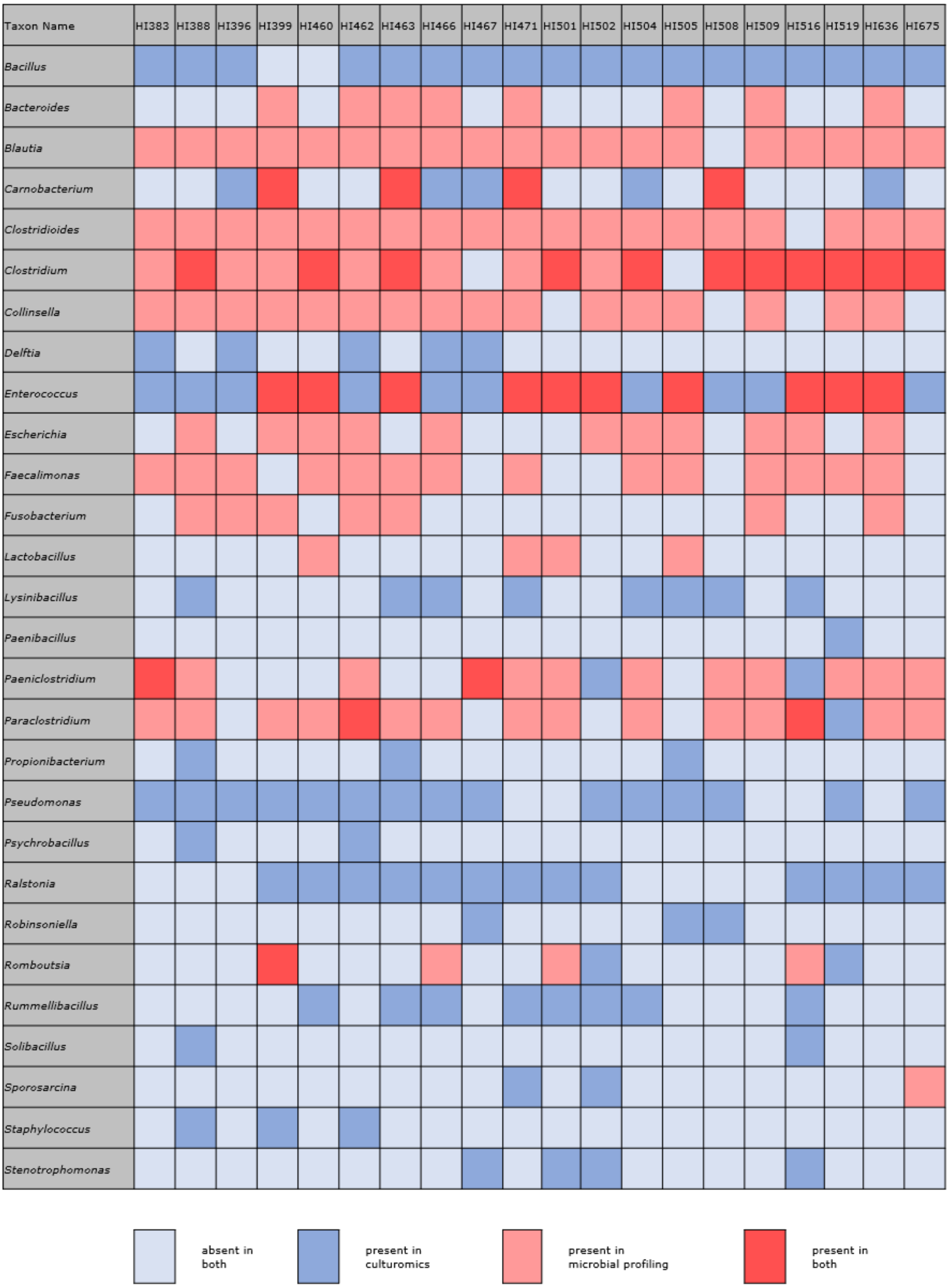

Supplementary Figure 5: Genera distribution detected by each methodological approach per faecal sample.

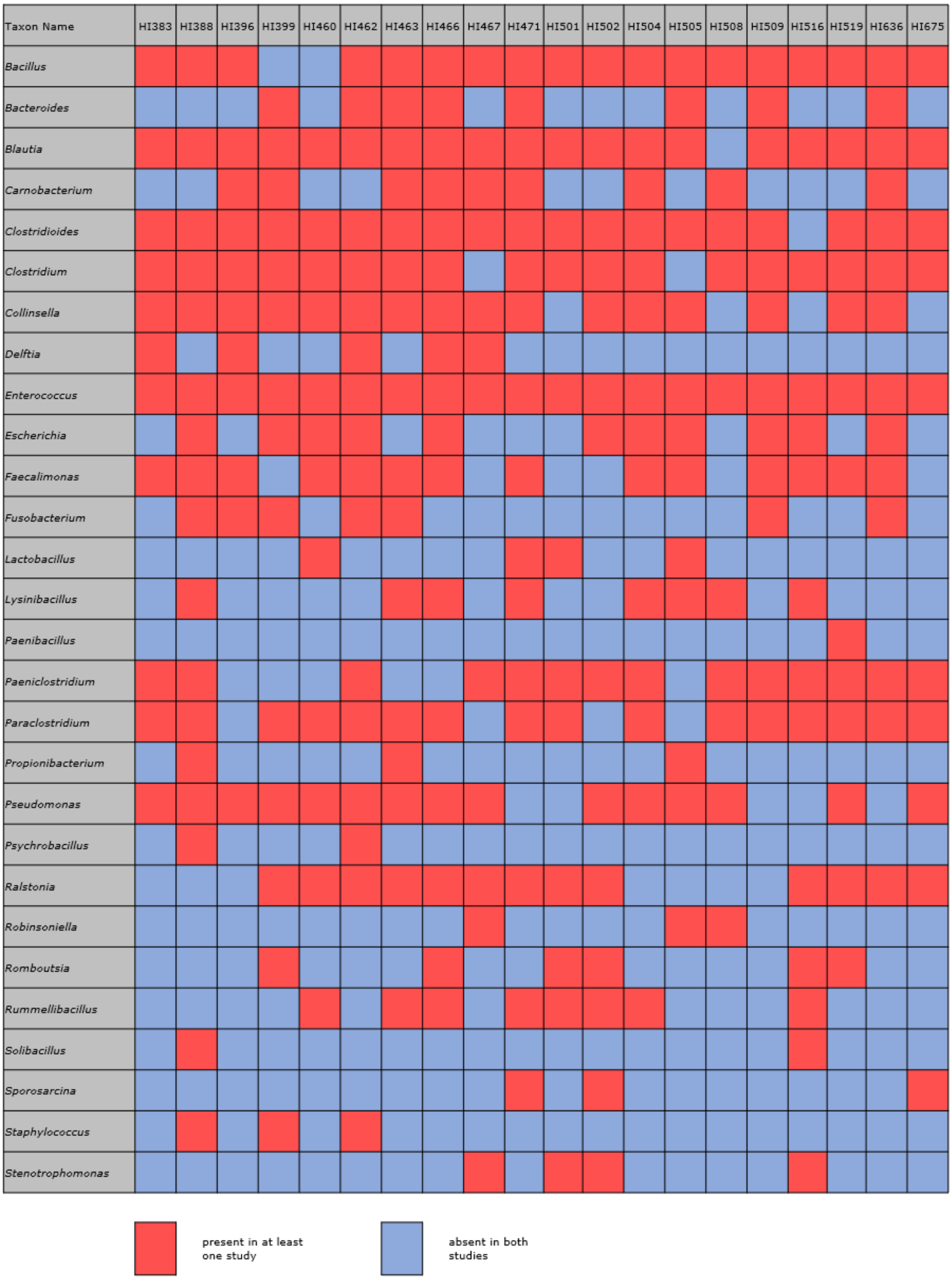

Supplementary Figure 6: Genera distribution per faecal sample.

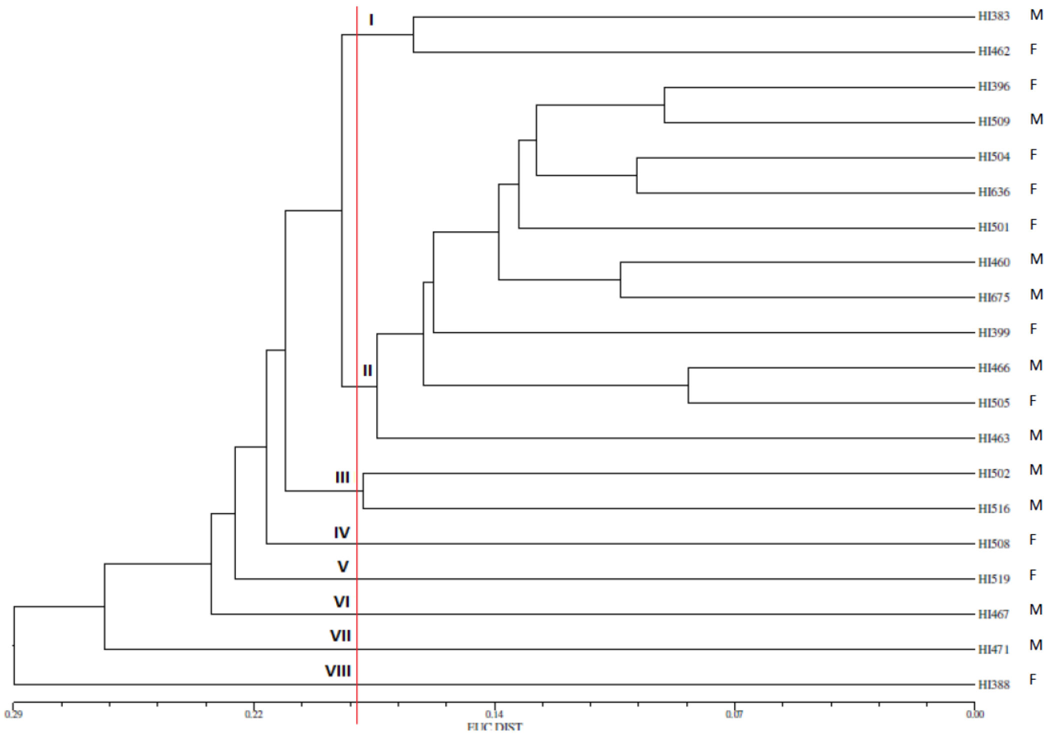

Supplementary Figure 7 – Dendrogram representing the relationship between the Egyptian mongoose specimens in terms of microbiota. The 20 specimens were clustered using the normalized Euclidean distance derived from the projection matrix of the PCA obtained from the microbiota data and clustered using UPGMA. The cut-off value was determined at 0.18 of normalized Euclidean distance, producing 8 clusters, 5 of them single member clusters. M – Male; F- Female.
